# Simple biomarkers to distinguish Parkinson’s disease from its mimics in clinical practice: a comprehensive review and future directions

**DOI:** 10.3389/fneur.2024.1460576

**Published:** 2024-09-19

**Authors:** Andrea Quattrone, Mario Zappia, Aldo Quattrone

**Affiliations:** ^1^Neuroscience Research Center, University “Magna Graecia”, Catanzaro, Italy; ^2^Institute of Neurology, Department of Medical and Surgical Sciences, Magna Graecia University, Catanzaro, Italy; ^3^Department of Medical, Surgical Sciences and Advanced Technologies, GF Ingrassia, University of Catania, Catania, Italy

**Keywords:** Parkinson’s disease, biomarkers, clinical practice, electrophysiology, MRI

## Abstract

In the last few years, a plethora of biomarkers have been proposed for the differentiation of Parkinson’s disease (PD) from its mimics. Most of them consist of complex measures, often based on expensive technology, not easily employed outside research centers. MRI measures have been widely used to differentiate between PD and other parkinsonism. However, these measurements were often performed manually on small brain areas in small patient cohorts with intra- and inter-rater variability. The aim of the current review is to provide a comprehensive and updated overview of the literature on biomarkers commonly used to differentiate PD from its mimics (including parkinsonism and tremor syndromes), focusing on parameters derived by simple qualitative or quantitative measurements that can be used in routine practice. Several electrophysiological, sonographic and MRI biomarkers have shown promising results, including the blink-reflex recovery cycle, tremor analysis, sonographic or MRI assessment of substantia nigra, and several qualitative MRI signs or simple linear measures to be directly performed on MR images. The most significant issue is that most studies have been conducted on small patient cohorts from a single center, with limited reproducibility of the findings. Future studies should be carried out on larger international cohorts of patients to ensure generalizability. Moreover, research on simple biomarkers should seek measurements to differentiate patients with different diseases but similar clinical phenotypes, distinguish subtypes of the same disease, assess disease progression, and correlate biomarkers with pathological data. An even more important goal would be to predict the disease in the preclinical phase.

## 1 Introduction

Parkinson’s disease (PD) is a common neurodegenerative disorder characterized by accumulation of intraneuronal misfolded alpha-synuclein aggregates and death of dopaminergic neurons in the substantia nigra (1, 2). The clinical diagnosis of PD is based on the presence of core motor signs, such as bradykinesia, rigidity and rest tremor, in the absence of clinical features suggestive of other diseases (2). The correct clinical diagnosis of Parkinson disease (PD) is often challenging, and several studies have demonstrated that the diagnostic accuracy varies considerably according to the disease duration and the expertise of the clinician, with high rates of misdiagnosis especially in early-stage patients and in primary-care centers (3–5). Based on the heterogeneity of clinical motor symptoms, two main PD subtypes have been described: Tremor-dominant PD (TD-PD) and akinetic-rigid or postural instability gait disorder (PIGD), showing several clinical and imaging differences (6–14). TD-PD patients often present with isolated rest tremor of a limb at the beginning of the disease, developing other parkinsonian signs later on; in these patients the clinical differential diagnosis may be difficult especially in the early stage of the disease, and the main alternative diagnoses other than TD-PD include essential tremor with resting tremor (ET plus), dystonic tremor, drug-induced tremor, and functional movement disorders (4, 15–18). On the other hand, the most common misdiagnoses in patients with akinetic-rigid PD are atypical parkinsonism, such as mainly progressive supranuclear palsy (PSP), multiple system atrophy (MSA) and cortico-basal degeneration (CBD), which share with PD several clinical features (3, 19–21). An accurate PD diagnosis is extremely important for prognostic implications (with better prognosis in non-parkinsonian tremor syndromes and worse prognosis in atypical parkinsonisms than in PD), but also for the future possibility to offer patients therapeutic approaches with disease-modifying drugs targeting specific molecular substrates, currently under investigation.

For this reason, in the last years, a plethora of studies focused on the development of biomarkers for differentiation between Parkinson’s disease and its mimics (20, 22–24), with most studies focusing on the differentiation between PD and atypical parkinsonism. The importance of developing biomarkers able to accurately differentiate PSP and MSA from PD has been further increased after the identification of the milder forms of these diseases termed PSP-parkinsonism (PSP-P) and MSA-parkinsonism (MSA-P), which have a clinical picture mainly characterized by rigidity and bradykinesia, strongly resembling PD (25–27). A few studies have also investigated the role of biomarkers in distinguishing patients with TD-PD from those affected by ET with rest tremor (ET plus) and other non-parkinsonian rest tremor syndromes. The field of biomarkers is rapidly expanding, with research progressing in multiple directions. Recent efforts have been focused on the development of new fluid or imaging biomarkers to support PD diagnosis, to predict the disease before symptom onset and to stratify patients based on the underlying molecular alterations (28). However, significant work remains to standardize these innovative techniques and facilitate the transition of new biomarkers from bench to bedside, integrating them into clinical practice. Concurrently, recent efforts are directed toward validating consolidated biomarkers in larger cohorts ensuring generalizability, and identifying simple biomarkers for widespread clinical use. Several biomarkers have been proposed to date, all showing high accuracy in differentiating PD from its mimics, but most of them have limited feasibility and usefulness in routine clinical practice (20, 29, 30). Some recent biomarkers are invasive (i.e., cerebrospinal fluid analysis), while others require specific MR sequences/PET radiotracers, or are based on multimodal imaging approaches/machine learning procedures which require high technology and complex data analysis usually performed by engineers rather than clinicians. Dopamine imaging (DaTSCAN) is a useful technique to support PD clinical diagnosis, but it is expensive and not widely available.

In this review, we considered the balance between accuracy and complexity of proposed techniques, and we focused on the biomarkers which met the minimum threshold of 80% sensitivity and specificity in previous studies, which are currently not included in the diagnostic criteria for PD (2) and are simple enough to be performed in clinical practice for differentiating PD from its mimics. We also discussed, for each biomarker, its usefulness in different clinical scenarios based on PD clinical presentation (TD-PD versus other tremor syndromes or PD akinetic-rigid versus atypical parkinsonisms) and highlighted possible future directions and perspectives.

## 2 Electrophysiological biomarkers

Several studies investigated the role of electrophysiological parameters in differentiating patients with PD from those with non-parkinsonian tremors or atypical parkinsonism (Table 1). The most investigated electrophysiological parameter was the blink reflex recovery cycle (BRrc), a measure for detecting brainstem interneuron hyperexcitability. In addition to the BRrc, some studies have investigated the usefulness of several electrophysiological features of rest tremor (RT) in distinguishing between TD-PD and other resting tremulous disorders, with the tremor pattern seeming the most valuable electrophysiological measure for the differential diagnosis of RT.

**Table 1:** Simple biomarkers to distinguish Parkinson’s disease from its mimics in clinical practice.

Biomarker	Category	Main role	Advantages	Limitations	Evidence
R2-BRrc	Electrophysiology	-Distinguishing early de-novo PD from aging -Distinguishing tremulous PD from essential tremor -Distinguishing PD from PSP or MSA (asymmetry index)	-quantitative -objective -low-cost -fast -non-invasive	-affected by medications (especially benzodiazepines) -not specific of PD (i.e., dystonia, NPH)	Evidence based on small single-centre studies
RT pattern/phase	Electrophysiology	-Distinguishing tremulous PD from other rest-tremor syndromes (essential tremor plus, dystonic tremor, drug-induced tremor)	-low-cost -fast -reproducible -non-invasive -quantitative (phase)	-pattern evaluation based on subjective assessment (minimal expertise required). Phase is quantitative but requires specific processing for calculation -qualitative binary response (Pattern alternating or synchronous)	Evidence based on a few large single-centre studies
SN hyperechogenicity	Imaging	-Distinguishing PD from aging -Distinguishing tremulous PD from essential tremor	-useful in the prodromal stage -quantitative -non-invasive	-not useful for distinguishing between PD and atypical parkinsonism -operator-dependent subjective evaluation -inadequate bone window in around 15% of patients	Evidence based on large studies and meta-analyses
Nigrosome-1 sign	Imaging	-Distinguishing PD from aging -Distinguishing tremulous PD from essential tremor and drug-induced parkinsonism	-useful in the prodromal stage (RBD) -non-invasive	-require MRI with field strength of 3 T or above and specific sequences not routinely performed in all centres -not useful for distinguishing between PD and atypical parkinsonism -based on subjective assessment (expertise required) -qualitative (yes/no)	-Evidence based on large studies and meta-analyses for PD vs. healthy subjects -Evidence based on small studies for PD versus other diseases
Qualitative MRI signs	Imaging	-Distinguishing between PD and atypical parkinsonism	-performed on routine MR images -non-invasive -high specificity (PSP vs. PD; MSA vs. PD)	-low sensitivity for PSP/MSA in the early stage of the diseases -qualitative, based on subjective assessment	-Evidence based on meta-analyses for putaminal hypointensity -Evidence based on a few large single-centre studies for hummingbird sign, morning glory signs and hot cross bun sign -Evidence based on small studies for the other signs
MRI measures	Imaging	-Distinguishing between PD and atypical parkinsonism	-performed on routine MR images -quantitative	-based on manual assessment (expertise required)	-Evidence based on a large multicentre study for the third ventricle width -Evidence based on a few large single-centre studies for midbrain and pons diameters -Evidence based on small studies for the other measures

BRrc, blink reflex recovery cycle; PD, Parkinson’s disease; PSP, progressive supranuclear palsy; MSA, multiple system atrophy; NPH, normal pressure hydrocephalus; RT, rest tremor; SN, substantia nigra; RBD, REM behavior disorder.

### 2.1 Blink reflex recovery cycle

**Figure 1:**
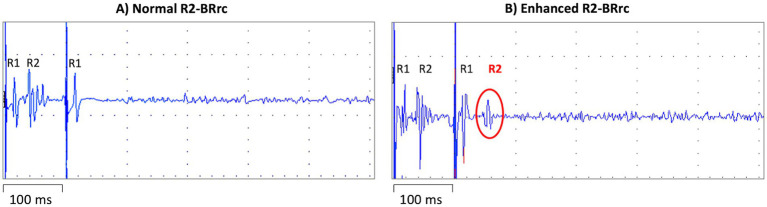
Blink reflex recovery cycle recording elicited by repeated stimulation of the supraorbital branch of the trigeminal nerve with two electrical stimuli separated by an inter-stimulus interval (ISI) of 100 msec. On the left **(A)**, a normal blink reflex recovery cycle recording, with R1 response following each stimulus and physiological inhibition (absence) of the second R2 response, due to the short ISI. On the right **(B)** a blink reflex recovery cycle recording in a patient with *de novo* Parkinson’s disease; the R2 response is clearly visible after the second stimulus delivered despite the short ISI, reflecting brainstem hyperexcitability.

Many studies investigated the usefulness of the R2 component of the BRrc (R2-BRrc) in parkinsonism or dystonia. The R2-BRrc is an electrophysiologic measure of brainstem excitability which can be easily performed using an electromyograph. The blink reflex can be elicited by stimulating the supraorbital branch of the trigeminal nerve and is composed by an early, homolateral response (Rl) followed by a late, bilateral response (R2) (31, 32). When the R2 response of the BRrc is evocated twice by two electrical stimuli of equal intensity, the second R2 is strongly inhibited with short inter-stimulus intervals (ISIs) of 100–200 msec, and gradually recovers with longer ISIs (500–700 msec) (31–33). In some pathological conditions including PD, the R2 response is present also at very short ISIs, reflecting brainstem hyperexcitability (31, 33, 34), as shown in Figure 1. The main limitation of this test is that several disorders including PD, atypical parkinsonism, normal pressure hydrocephalus, drug induced parkinsonism and dystonia show enhanced R2-BRrc response, thus making the brainstem hyperexcitability of unspecific value (34–42). On the other hand, R2-BRrc is a simple and widely available technique which can be useful as screening test for distinguishing tremulous PD or dystonic tremor from essential tremor, and healthy subjects (39, 43, 44).

#### 2.1.1 PD versus control subjects

Some studies showed an increased R2 component of the blink reflex recovery cycle in PD patients compared to normal subjects (31, 34, 42, 44), and these abnormalities were even greater in patients with advanced PD (42) or with lateral trunk flexion (35). In PD patients, the enhancement of R2-BRrc response was observed in OFF-state, while this response was like that observed in normal subjects in ON-state, suggesting that it may be related to the central dopamine activity and may be influenced by dopaminergic therapy (34). For this reason, R2-BRrc should be used as diagnostic biomarker mainly in *de novo* PD patients, or in PD patients with long-lasting drug withdrawal to avoid the interference of dopaminergic therapy (34, 44). The first study in *de novo* tremor-dominant PD patients was carried out by Nisticò et al. (44), showing that R2-BRrc response was highly enhanced in *de novo* PD patients at all investigated ISIs (100–750 ms) in comparison with control subjects, who showed no R2-BRrc response at ISIs of 100–200 msec and a detectable but lower R2-BRrc response at ISIs ≥300 msec (44). R2-BRrc might also be used for differentiating organic rest tremor (i.e., due to Parkinson’s disease) from functional tremors. At the present time, R2-BRrc has been reported to accurately distinguish organic from functional blepharospasm (40) but no data exist on the usefulness of R2-BRrc for differentiation of parkinsonian tremor from functional tremor. It is possible to hypothesize that functional neurological disorders have normal brainstem excitability, but further studies to clarify the role R2BRrc in functional tremor are warranted.

#### 2.1.2 PD versus essential tremor with or without rest tremor

The R2-BRrc has been reported to accurately differentiate patients with tremulous PD from those with ET. In PD patients, the R2-BRrc response was enhanced at all investigated ISIs (100–750 ms) while ET patients showed normal R2-BRrc values at all ISIs (44, 45). Of importance, the R2-BRrc has also been reported to differentiate *de novo* PD from ET with rest tremor, recently classified as ET plus (44). Both diseases showed enhanced R2-BRrc at ISIs ≥150 msec, having higher brainstem excitability than “pure” ET patients (44, 46), but only *de novo* PD patients showed enhanced R2-BRrc response at a very short ISI of 100 msec, showing a sensitivity and specificity of 100% in differentiating PD from ET with rest tremor (44). Further studies in larger cohorts of *de novo* tremor-dominant PD and ET with rest tremor patients are warranted to validate these findings.

#### 2.1.3 PD versus neurodegenerative atypical parkinsonism

A few studies investigated the R2-BRrc in atypical neurodegenerative parkinsonism, demonstrating that the R2-BRrc response was pathologically enhanced in PD, PSP and MSA patients while it was normal in patients presenting with CBS (37, 47, 48). Indeed, the presence of an early recovery of the R2-BRrc differentiated PSP from CBS with a specificity and sensitivity of 87.5 and 91.7%, respectively (48). A recent study investigated whether the R2-BRrc could distinguish between PD and atypical parkinsonism when evaluated on both sides. Sciacca and colleagues (49) reported that *de novo* PD patients had an increased brainstem excitability by stimulating in the clinically less affected side compared with most affected side, while this asymmetry of R2-BRrc was not found in parkinsonism such as PSP or MSA. In this preliminary study, the asymmetry index of R2-BRrc response [calculated as (Side1–Side2)/(Side1 + Side2)] showed sensitivity above 85% and specificity above 90% in differentiating patients with *de novo* PD from those with PSP or MSA (49). These promising results, however, need confirmation and validation in larger studies.

#### 2.1.4 PD versus secondary parkinsonism

There is evidence that patients with normal pressure hydrocephalus (NPH) may show parkinsonian signs and radiologic overlap with progressive supranuclear palsy, including hummingbird sign, reduced midbrain area and third ventricle enlargement (50–53). A recent study (38) showed that R2-BRrc response was enhanced in patients with NPH in comparison with control subjects, probably due to the brainstem compression by ventricular dilatation, and no differences were found between NPH and *de novo* PD patients. In addition to NPH, R2-BRrc response was also enhanced in patients with drug-induced parkinsonism (DIP), though with a slight significant difference between DIP and *de novo* PD patients at ISI 100 msec (54). No data are currently available on R2-BRrc in vascular parkinsonism. Based on the available evidence, the R2-BRrc seems not suitable to differentiate PD from secondary parkinsonism, and further studies evaluating the asymmetry index of R2-BRrc are warranted.

Overall, evidence suggests that PD patients show brainstem excitability as documented by R2-BRrc in comparison to normal subjects and patients with classical essential tremor while preliminary data point at a possible differentiation of TD-PD from ET with rest tremor by focusing on ISI of 100 msec. Of note, the R2-BRrc did not accurately differentiate PD from atypical or secondary parkinsonism, suggesting the possible usefulness of asymmetry index to differentiate PD patients from other parkinsonian disorders.

### 2.2 Electrophysiological analysis of rest tremor

Rest tremor (RT) is a type of involuntary rhythmic movement that occurs when a person is at rest and not voluntarily engaging in any activity (2, 55). RT is typical of Parkinson’s disease and occurs in approximately 75% of PD patients (56, 57), but this sign can also be observed in other neurological disorders. There is evidence that 18–88% of patients with ET display a rest tremor (rET) (58–61) and these patients are now classified as ET plus (55). Drug-induced parkinsonism (DIP) has been associated with use of drugs blocking dopamine receptors or some antiepileptic drugs, such as valproic acid, and often presents with tremor at rest (62, 63). Moreover, RT can be observed in atypical parkinsonism, dystonic tremor, scans without evidence for dopaminergic deficit (SWEDD), and psychogenic tremor (15, 64–70). Differentiating tremulous PD from non-parkinsonian RT syndromes is clinically challenging in the absence of overt bradykinesia and rigidity, and often requires the single photon emission computed tomography with 123I-ioflupane (DaTscan) (17, 71). In addition, the electrophysiological tremor analysis has gained a growing importance in the diagnostic work-up of RT, considering that it is a cheap and widely available procedure (72, 73). Several authors evaluated the electrophysiological features of rest tremor in PD and other tremor syndromes, and the most investigated parameters were frequency, amplitude, burst duration and pattern of tremor (74–76). Tremor analysis is classically performed with accelerometry or surface electromyography (sEMG). Accelerometers mainly provide information on tremor frequency and amplitude, while sEMG also detects burst duration and muscular contraction pattern (74–77).

#### 2.2.1 Tremor dominant PD (TD-PD) versus rest tremor syndromes with integrity of dopaminergic system

**Figure 2:**
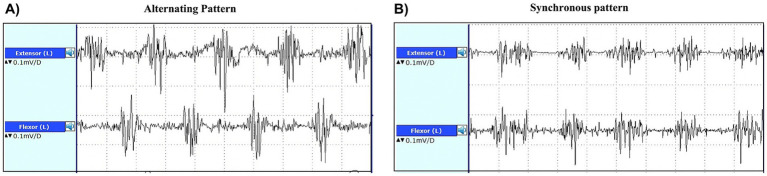
EMG recordings from the extensor carpi radialis and flexor carpi ulnaris muscles of a patient with alternating pattern of rest tremor **(A)** and a patient with synchronous pattern of rest tremor **(B)**. In the trace on the left, the bursts of antagonist muscles are shifted (alternating pattern), while in the trace on the right side of the figure, the forearm antagonist muscle contract at the same time (synchronous pattern).

In TD-PD patients, the rest tremor is characterized by a frequency of 4–7 Hz and is often asymmetric at the beginning of the disease (2, 55, 56). The movement initiation typically suppresses rest tremor, but it can re-emerge when a posture is sustained (re-emergent tremor) (57, 78, 79). A plethora of studies investigated the electrophysiological features of rest tremor in PD patients and postural tremor in ET patients. On the other hand, a lower number of studies investigated the usefulness of EMG tremor analysis in differentiating parkinsonian RT from other non-parkinsonian rest tremor syndromes (80–82). These studies showed that parkinsonian rest tremor is often characterized by higher amplitude and slightly lower frequency and burst duration than other rest tremors, but values showed large overlap among different diseases, do not allowing to distinguish parkinsonian tremor from non-parkinsonian RT disorders at the individual level (55, 80–83). Tremor amplitude shows high variability among patients and can be influenced by stress, mental concentration, and medications, making this tremor feature of limited value in the differential diagnosis of rest tremor. Similarly, tremor frequency is not very helpful in the diagnosis of tremulous disorders because it ranges from 4 to 8 Hz in most pathological tremors (55). Thus, the assessment of RT frequency could be mainly useful to rule out rare conditions such as myorhythmia, which may present as focal limb rhythmic movements at rest or during action and is characterized by a very low (1–3 Hz) frequency (55, 81, 84). In addition, the variability of tremor frequency during EMG recording, together with other clinical and electrophysiological features, can be supportive of psychogenic tremor (85, 86). Differently from amplitude and frequency, the muscular contraction pattern of RT seems a key electrophysiological feature for differentiating PD from other non-parkinsonian RT syndromes (82). The tremor pattern is commonly evaluated through visual evaluation of surface EMG recordings, usually performed by neurologists or expert technicians, and classified as synchronous (when antagonist muscles contract at the same time) or alternating (when the bursts of antagonist muscles are shifted), as shown in Figure 2 (74, 81, 87). While some studies involving surface EMG have identified both patterns in postural tremor of both PD and ET cases (88, 89), making this tremor feature of uncertain diagnostic significance in presence of active muscle contraction (action tremor), the pattern of rest tremor remains stable over time and is a reliable parameter to support the differential diagnosis of rest tremor syndromes (81–83). It is known since long ago that the typical rest tremor observed in PD shows an alternating pattern on EMG recordings (57, 90), but the usefulness of this tremor feature of RT has been systematically investigated only in the last years. Some pilot studies demonstrated that the pattern of RT accurately distinguished patients with tremor-dominant PD (alternating pattern of RT) from those affected by Essential Tremor with rest tremor (ET plus) (80) and from those with drug-induced RT showing synchronous pattern of RT (54). These preliminary results have been validated in a very recent study (82) carried out in a large cohort of 205 consecutive patients presenting with RT, demonstrating that RT pattern was able to accurately predict the DaTscan result and thus to help distinguish between parkinsonian and non-parkinsonian rest tremors. More in detail, 91% of RT patients with alternating pattern showed a striatal dopaminergic deficit, while 79% of patients with synchronous rest tremor had normal DaTscan, thus significantly outperforming other RT electrophysiological features which showed larger overlap between DaT+ and DaT- rest tremor patients and were not able to predict the dopaminergic deficit in RT patients (82). The visual assessment of RT pattern is straightforward in most cases, but it requires some expertise in tremor analysis. For a quantitative and objective evaluation, the temporal relationship between antagonistic muscles can also be assessed through the calculation of tremor phase (91, 92). High tremor phase values (typically >90°) reflect a shift between contraction bursts of antagonist muscles and thus correspond to an alternating pattern on EMG recordings, while phase values lower than 90° correspond to a synchronous pattern (91, 93). Tremor phase calculation can be performed using cross-spectral analysis on EMG tremor recordings (83, 91); in addition, we have recently developed and validated a new wearable mobile tool for the automated phase displacement calculation in ambulatory settings (94), which showed very good agreement with the gold standard phase calculation techniques in a sample of 21 subjects (14 PD patients with alternating RT pattern and 7 ET plus patients with synchronous RT pattern). This portable wearable mobile device, termed μEMG, is a wrist watch-like support with surface electrodes that record the contraction of the antagonist muscles in the forearm; a mobile application provides the tremor phase calculation (94). Finally, tremor phase/pattern can be estimated by using modern machine learning technology based on inertial tremor data acquired through accelerometers and gyroscopes (95), thus paving the way for new small and easy devices to be used for RT differential diagnosis in large populations in clinical routine. Limitation of current research in the field include the monocenter design of available studies, often carried out in referral centers for tremor. Thus, future research may include further technological advancement, international validation studies to confirm the diagnostic potential of tremor pattern/phase in distinguishing PD from other rest tremor syndromes in clinical settings, also in population which have been under-represented in current research, and provide a deeper focus on non-parkinsonian RT syndromes other than ET plus, including larger cohorts of dystonic, drug induced or functional tremor patients.

#### 2.2.2 TD-PD versus essential tremor-Parkinson’s disease (ET-PD) syndrome

Essential tremor-Parkinson’s disease (ET-PD) is a syndrome characterized by the occurrence of PD in patients with a previous history of ET. Although some authors proposed that co-occurrence of ET and TD-PD in the same individual might be coincidental, others suggested that there is an actual association between these two diseases (96–98). To date, only few studies investigated the electrophysiological features of rest tremor in ET-PD patients and there are conflicting results (99, 100). One study reported that ET-PD patients displayed a synchronous pattern of rest tremor, allowing to differentiate TD-PD from ET-PD and suggesting that in these patients the RT is like that occurring in ET plus (synchronous pattern) rather than that typical of TD-PD (alternating pattern) (100). On the other hand, another study showed that ET-PD patients had a synchronous pattern of RT in 31%, an alternating pattern in 50% and a mixed pattern in the remaining 19% of cases (99), thus more data are needed to establish the pattern of RT in ET-PD patients. It is possible that different patterns may suggest different pathophysiological bases of RT in these patients, and future studies May clarify this point.

#### 2.2.3 PD versus atypical parkinsonism

Rest tremor occurs less often in atypical parkinsonism than in PD, although cases with a RT as the initial presenting sign have been reported; moreover, RT usually lacks the classical “pill-rolling” aspect which is typical of PD (15, 68–70). According to a large pathological study the prevalence of RT is around 20% in PSP patients (70), and no study focused on the RT electrophysiological features to differentiate between PSP and PD. PSP-P patients, who usually show a clinical phenotype resembling PD, might have a higher prevalence of RT, but studies systematically investigating this hypothesis in large cohorts of patients are still missing. Rest tremor is more common in MSA than in PSP patients, with a frequency ranging from 32 to 44% in MSA-P and from 17 to 26% in MSA-C (68, 69). No systematic data on RT pattern in atypical parkinsonism are currently available. A very recent study (101) proposed the use of RT electrophysiological features for distinguishing between PD and MSA. While the frequency was not different between these two disorders, the presence of harmonics in RT recordings, defined as additional frequency peaks at integer multiples of the main tremor frequency peak, showed 71% sensitivity and 95.5% specificity in distinguishing PD from MSA-P patients (101). These findings are novel and interesting but yet very preliminary and maybe more suitable for research purposes than for clinical practice, due to method complexity.

## 3 Transcranial sonography of substantia nigra

**Figure 3:**
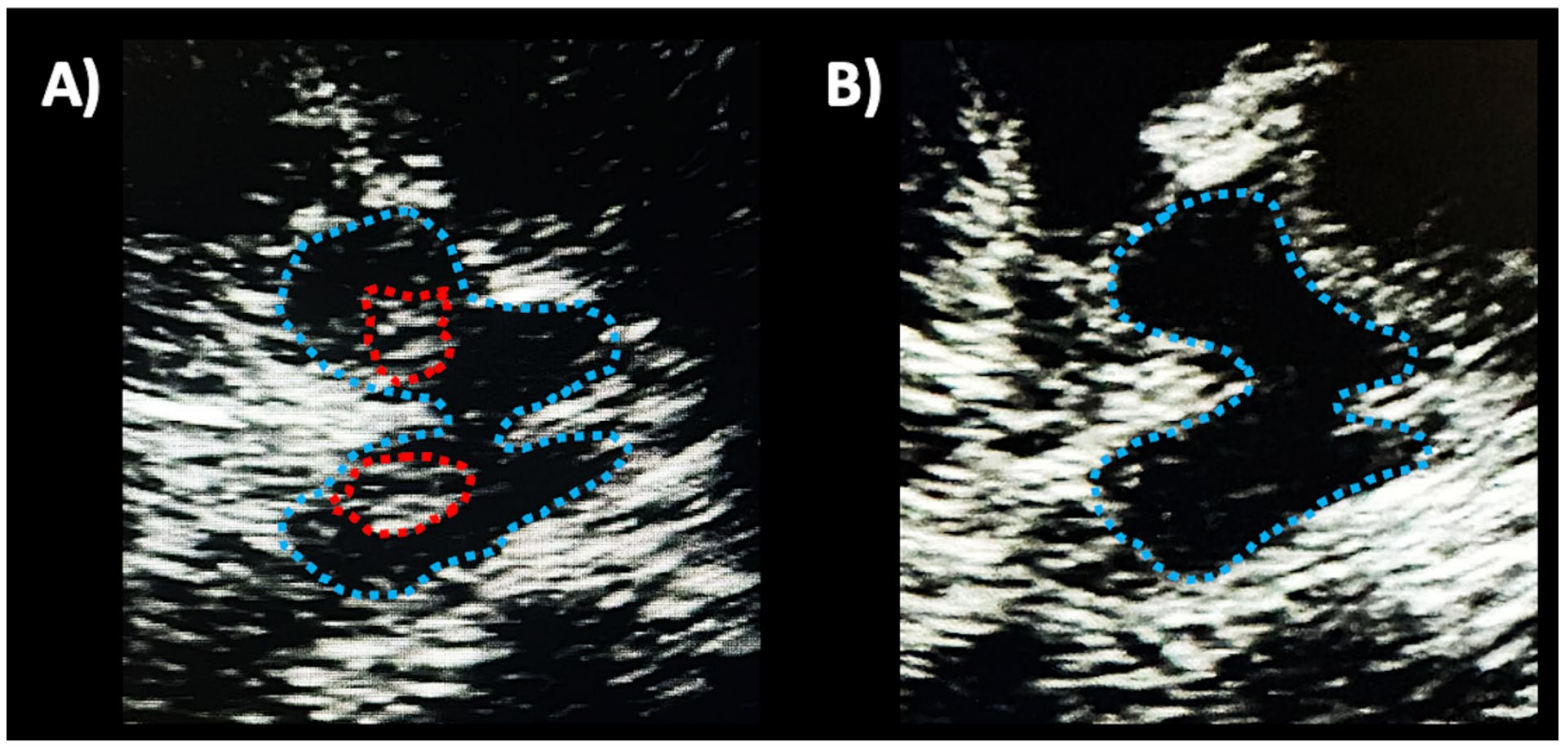
Transcranial sonographic images showing an axial view of the midbrain in a patient with Parkinson’s disease **(A)** and in a healthy subject **(B)**. In both images, the midbrain is delimited by a light blue dotted line. In the left image, hyperechogenic (bright) areas are visible bilaterally within the substantia nigra, encircled by red dotted lines. On the contrary, the midbrain is homogenously dark in the right image, reflecting normal echogenicity.

Transcranial sonography (TCS) is a non-invasive, low-cost, available technique to support the diagnosis of PD. This technique allows to investigate the echogenicity of the substantia nigra (SN) through a temporal bone window, and the presence of hyperechogenic (bright) areas May reflect increased iron deposition in the SN (102), as shown in Figure 3. If hyperechogenic areas are visible within the SN, these can be manually encircled, and the total area can be measured (103, 104).

### 3.1 PD versus healthy subjects

In 1995, Becker and colleagues first described hyperechogenicity of the substantia nigra with transcranial ultrasound midbrain imaging in patients with Parkinson’s disease compared with controls (105). Several independent studies have confirmed the presence of hyperechogenic areas within the SN larger (usually >0.20 cm^2^) than those observed in healthy subjects in up to 90% of PD patients (104, 106–110), especially contralaterally to the side with the more severe symptoms (107, 109, 111). Several systematic review and meta-analyses were conducted to evaluate the diagnostic accuracy of substantia nigra hyperechogenicity by TCS for the diagnosis of PD. Li et al. (112) showed good overall diagnostic accuracy of TCS in differentiating PD from normal controls, with a pooled sensitivity of 0.83 (95% CI: 0.81–0.85) and a pooled specificity of 0.87 (95% CI: 0.85–0.88). These results were confirmed by a more recent meta-analysis conducted by Tao et al. (113), who analyzed 39 studies including 3,123 patients with PD. The authors found that the pooled sensitivity and specificity of TCS were 0.84 (95%; 0.81–0.87) and 0.85 (0.80–0.88), respectively, for differentiating PD from healthy controls. Finally, Xu et al. (110) in a large cohort of PD and healthy subjects found that AUC for differentiation of PD patients from healthy controls was 0.92 and 0.91 and optimal cut-off values of 0.20 and 0.21 cm^2^ derived from the assessments performed by two different readers, respectively. While most studies found TCS useful for distinguishing PD patients from controls, mixed evidence exists on the role of TCS as biomarker of disease severity in PD. The size of hyperechogenic areas was associated with disease severity in some studies (109, 114) but not in others (104, 106, 108, 111, 115). However, several possible confounding factors need to be considered. For example, SN hyperechogenic areas seem larger in akinetic-rigid PD than in TD-PD (114, 116, 117), and one large study by Zhou et al. (116) found that the size of SN echogenicity was correlated with disease duration in Chinese patients with PD who were male and non-TD subtype, but not in females and other motor subtypes. On the contrary, Sheng et al. found that SN hyperechogenic did not correlate with disease severity in any PD subtype (117). Overall, the correlation between the size of SN echogenicity and clinical PD severity needs further investigations.

Of importance, evidence exists that SN hyperechogenicity may represent a risk factor for PD, and it is included among risk markers in the MDS Research Criteria for Prodromal Parkinson’s Disease (118). A study (119) in a very large cohort of 1,260 PD-free individuals ≥50 years who underwent a 5 year follow-up assessment, showed that hyperechogenic substantia nigra was the most frequent baseline sign in individuals developing PD after 3 years (80.0%) and 5 years (85.7%) compared to healthy controls (17.5%), with higher sensitivity than clinical prodromal signs such as hyposmia or mild parkinsonian signs. Participants with SN hyperechogenicity at baseline had a more than 20.6 times increased risk to develop PD in this time span (5 years) than those without this echo feature (119). Similar results on the superiority of TCS to these clinical features were reported in another population prospective study (120) comparing different potential risk factors such as SN hyperechogenicity, olfactory function and mild parkinsonian signs, in incident PD identified at 5 year and 10 year follow-up visits. Moreover, the risk of incident was similar at 5 and 10 year follow-up for hyposmia patients, while it was higher within the first 5 years for subjects with SN hyperechogenicity (120). On the contrary, TCS of the substantia nigra is not useful to identify subjects with idiopathic REM sleep behavior disorder (RBD) at risk of conversion to PD within 5 years of follow-up (121, 122).

### 3.2 PD versus atypical parkinsonism

Studies with TCS in patients with atypical parkinsonism showed that hyperechogenicity of the SN was less commonly observed in MSA-P and PSP patients than in PD patients (104, 123–126); on the other hand, there is some preliminary evidence of SN hyperechogenecity as a common finding in CBS patients (104, 127, 128) and Lewy Body Dementia patients (129). Among the largest studies, Zhou et al. (130) demonstrated that TCS discriminated PD from MSA with sensitivity of 74.1% and specificity of 61.6%, and Alonso-Canovas et al. (131) reported a 80% sensitivity and 61% specificity in distinguishing PD from atypical parkinsonism. Overall, large meta-analyses showed that TCS had 75–85% pooled sensitivity and 69–71% pooled specificity to discriminate PD from atypical parkinsonism (132–134), with slightly better performances in studies where a cutoff of 20 mm^2^ was employed (133), and 86% sensitivity (95% CI 75–86%) and 70% specificity (95% CI 56–81%) to differentiate PD from PSP patients (135). Much more challenging is the differentiation of PSP-P from PD using TCS, since most (70–80%) PSP-P patients had hyperechogenic SN (136–138), making other biomarkers necessary for this classification task.

### 3.3 PD versus secondary parkinsonism

A few studies investigated SN echogenicity in secondary parkinsonism. As for the differential diagnosis between PD and drug-induced parkinsonism (DIP), two studies (139, 140) demonstrated that TCS had sensitivity of 75–81.2% and specificity of 84.1–91.1% in distinguishing patients presenting with clinically persistent or aggravated parkinsonism after drug withdrawal (unmasked PD) from those recovering after drug withdrawal (DIP), and another study reported 88.2% sensitivity and 87.5% specificity in distinguishing PD from DIP (141). Overall, these data suggest that DIP have normal SN echogenicity in most cases. A couple of studies demonstrated that 80% of patients with vascular parkinsonism had normal SN echogenicity, with TCS showing 85–90% sensitivity and 80% specificity in distinguishing PD from vascular parkinsonism (142, 143). Finally, evidence of SN hyperechogenicity exist also in other disease which May manifest with parkinsonian symptoms, such as Wilson’s disease (40–50% of patients with neurologic impairment showing SN hyperechogenicity) (144, 145), and a few reports in Normal pressure hydrocephalus (129) and Perry syndrome (146), suggesting that this finding is not entirely pathognomonic for PD.

### 3.4 PD versus non-parkinsonian tremor syndromes

Several studies investigated the role of TCS in distinguishing PD from essential tremor. The existing evidence is summarized by a systematic review and meta-analysis including 1,264 PD and 824 ET patients (147) demonstrating that the pooled sensitivity and specificity for TCS in the differential diagnosis of PD versus ET was 84.6% (95% CI, 79.4–88.6%) and 83.9% (95% CI, 78.4–88.2%), respectively. These data suggest that this technique could be performed as a screening test to evaluate tremulous patients suspected of having PD who need to be examined with DaTscan. At the present time, however, no evidence exists on the possible role of TCS in distinguishing PD from ET plus patients showing resting tremor like that observed in PD. Further studies to clarify the possible usefulness of this technique for differentiation of PD from ET plus are warranted.

Overall, TCS May be used as an auxiliary diagnostic tool for Parkinson’s disease, especially in distinguishing PD from healthy subjects, ET patients, and drug-induced parkinsonism; lower accuracy has been reported in distinguishing PD from atypical parkinsonism, due to high false positive rates. The main advantages of TCS are its non-invasiveness, the low-cost and the potential large availability; the main limitation is that this technique is operator-dependent, with possible discrepancies among centers (132). Several studies demonstrated good inter-rater agreement when the SN assessment was performed by physicians with expertise in SN sonographic assessment, while poor agreement was found between raters with lower expertise (148, 149). Possible future advancement to increase generalizability and obtain more homogeneous measures across centres May include the development of software for automated assessment of SN hyperechogenic area (150), or higher standardization of SN hyperechogenic area calculation procedure (i.e., defining precise anatomical landmarks or normalization strategies). A final limitation is that this examination May be unsuccessful in about 10–20% of subjects, due to an inadequate bone window (103, 104).

## 4 Simple magnetic resonance imaging biomarkers

Conventional MRI of the brain shows very few abnormalities in PD, but it has a major role in differentiating PD from mimics allowing to rule out several diseases with similar clinical features, such as atypical parkinsonism, vascular parkinsonism, NPH and some rare disorders presenting with parkinsonism (151–153). Over the last few years many studies focused on the differentiation between PD and parkinsonism with MRI. Most of the proposed biomarkers, however, require specific sequences not commonly performed in clinical practice or complex quantitative data analysis usually performed by engineers rather than clinicians, and thus are prerogative of few centers owning the necessary expertise and technology. MRI biomarkers for the differential diagnosis between PD and other parkinsonism in clinical practice include mainly the qualitative evaluation of MR images and some simple measurements which can be performed directly on MR images by neurologists or neuroradiologists (Table 1).

The routine brain MRI protocol for patients presenting with parkinsonism usually includes: a three-dimensional (3D) T1-weighted sequences for evaluation of brainstem and basal ganglia atrophy; an axial T2-weighted sequences for evaluation of brain atrophy and signal changes in basal ganglia and brainstem structures; a 2D or 3D T2-weighted fluid-attenuated inversion recovery (FLAIR) for the evaluation of brain vascular lesions; a susceptibility sensitive sequence, either T2* or susceptibility weighted imaging (SWI), to evaluate the iron deposition in the basal ganglia and substantia nigra; a diffusion-weighted imaging (DWI) to evaluate acute vascular lesions and to exclude Creutzfeldt-Jakob disease in rapidly progressive parkinsonism (154, 155). In the future, the protocol May also include a Neuromelanin-sensitive T1-weighted sequence which has recently demonstrated to be useful for detecting the depigmentation of the substantia nigra occurring in PD patients, but this latter sequence is still not widely used in clinical practice (156, 157).

### 4.1 Qualitative MR imaging features

#### 4.1.1 The nigrosome-1 (swallow-tail sign)

##### 4.1.1.1 PD versus control subjects

**Figure 4:**
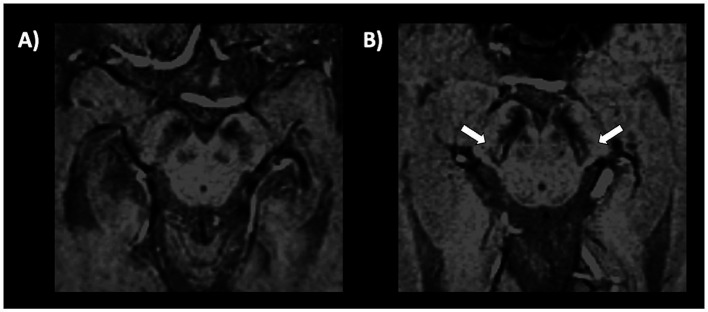
A 3T brain susceptibility weighted imaging (SWI) axial MR image showing the substantia nigra in a patient with Parkinson’s disease **(A)** and in a healthy subject **(B)**. The substantia nigra in the left image has a homogeneous dark appearance bilaterally, and the nigrosome-1 is not visible. Conversely, in the image on the right side of the figure, a small hyperintense area corresponding to the nigrosome-1 is visible in the dorsolateral part of the substantia nigra, bilaterally (swallow-tail sign present, which is the normal appearance).

The only qualitative sign described so far which can be observed on MR images in PD patients is the absence of nigrosome-1 hyperintensity on iron-sensitive T2* or susceptibility weighted imaging (SWI) MR images. The neurons of the pars compacta of the substantia nigra (SNc) are affected by the neurodegenerative process in PD, especially in the nigrosome-1, which is a small cluster of dopaminergic neurons located in the dorsolateral portion of SN. The appearance of nigrosome-1 was originally described using ultra-high magnetic field strengths of 7T-MRI (158, 159) but it can be evaluated also using the widely available 3T-MRI scanners (160–163). In heathy subjects, the nigrosome-1 appears on iron-sensitive T2*/SWI images as an hyperintense area with an ovoidal or “comma” shape in the dorsolateral part of the SNc surrounded by hypointense areas (SNc and medial lemniscus), resulting in an image which resembles the tail of a black swallow on axial images (termed “swallow-tail sign”), as shown in Figure 4 (159, 162, 164, 165). On the contrary, in PD patients, the degenerative process and increased iron deposition determine a loss of the nigrosome-1 hyperintensity on iron-sensitive T2*/SWI MR images, resulting in homogenous dark appearance of SN and thus absence of the swallow-tail sign (Figure 4) (162, 164, 165). Several different meta-analyses (160, 166–168) showed that the nigrosome-1 sign had sensitivity and specificity above 90% for differentiation of PD from healthy subjects. The absence of nigrosome-1 is commonly observed bilaterally in most PD patients; however, the unilateral or bilateral absence of dorsolateral nigral hyperintensity showed similar sensitivity and specificity for PD diagnosis, making both these findings equally valid (161). Moreover, several studies found strong associations between the nigrosome-1 sign and dopamine imaging results, pointing at the nigrosome-1 as a biomarker for nigrostriatal dopaminergic degeneration in Parkinsonism (162, 169, 170) and also in RBD patients (171–173).

##### 4.1.1.2 PD versus essential tremor

To date, no simple MRI biomarkers have been reported to differentiate tremulous PD from non-parkinsonian tremor syndromes. A few small studies pointed at the usefulness of the nigrosome-1 imaging in distinguishing PD patients from ET patients (174–176), but no large prospective studies have been carried out in this direction so far to validate the preliminary findings. Some authors (174) found in a small ET cohort (11 ET patients and 38 PD patients) that the absence of both nigrosomes-1 was 96% accurate to differentiate PD from ET patients, and others (176) reported 87.5% accuracy for at least unilateral nigrosome-1 absence in distinguishing between 16 PD and 16 ET patients. Moreover, the area of nigrosome-1 hyperintense signal has also been reported to be significantly smaller in PD (median = 2.1 mm^2^) than in ET patients (median = 8.3 mm^2^) (177). No data, however, exist about the presence/absence of nigrosome-1 on SWI in ET patients with rest tremor (ET plus) or other non-parkinsonian rest tremor syndromes (SWEDDs). Future studies are needed to clarify the possible role of nigrosome-1 in differentiating parkinsonian from non-parkinsonian rest tremor syndromes.

##### 4.1.1.3 PD versus atypical and secondary parkinsonism

The absence of nigrosome-1 hyperintensity on SWI it has been reported also in atypical parkinsonism, not allowing to distinguish between PD from other disorders with dopaminergic deficit.

A few small studies demonstrated that the nigrosome-1 loss can accurately differentiate atypical parkinsonism from healthy controls, with excellent sensitivity and specificity (178, 179). The unilateral o bilateral absence of nigrosome-1 in MSA and PSP patients, however, makes this finding not useful for differentiating between PD and other neurodegenerative parkinsonisms (179).

Two small studies (180, 181) showed absence of dorsolateral nigral hyperintensity at least in one side in most patients with Lewy Bodies Dementia (LBD), but also in some patients with frontotemporal dementia and Alzheimer’s disease, who usually do not show dopaminergic damage, raising some doubts on the specificity of this MRI sign. Considering bilateral loss, sensitivity decreased (53%) but specificity increased (82–100%). Further studies are needed to better investigate the presence of nigrosome-1 absence in dementia.

One study (182) in a small cohort of patients (PD 29 and DIP 20, respectively) showed that the absence of nigrosome-1 had 100% sensitivity and 85% specificity in differentiating PD from drug-induced parkinsonism (DIP). Only one study evaluated this MRI sign in vascular parkinsonism, reporting nigrosome-1 loss in around 15/19 (44.1%) patients (183). Overall, these findings suggest that this MR features May be useful for differentiating neurodegenerative parkinsonism (PD and atypical parkinsonian syndromes) from secondary parkinsonism, especially DIP, but these results, still needs validation in larger studies.

Overall, despite the heterogeneity of MR acquisition parameters, visual assessment of the nigrosome-1 loss seems an accurate biomarker to distinguish PD from healthy subjects also in the early stage of the disease. This radiological sign can be assessed on 7T or 3T-MR images, while poor diagnostic performances have been reported on 1.5T MR images (179). Regarding differential diagnosis, the unilateral o bilateral absence of nigrosome-1 did not differentiate PD from atypical parkinsonism but can help clinicians to distinguish PD from DIP. It is important to note that neither nigrosome-1 evaluation on MRI nor DaT scan is a standalone diagnostic test for parkinsonism. However, both these techniques provide valuable additional information that can support the diagnosis, help differentiate parkinsonian disorders and guide appropriate management and treatment decisions.

#### 4.1.2 Hummingbird, mickey mouse, and morning glory signs

##### 4.1.2.1 PD versus atypical parkinsonism

**Figure 5:**
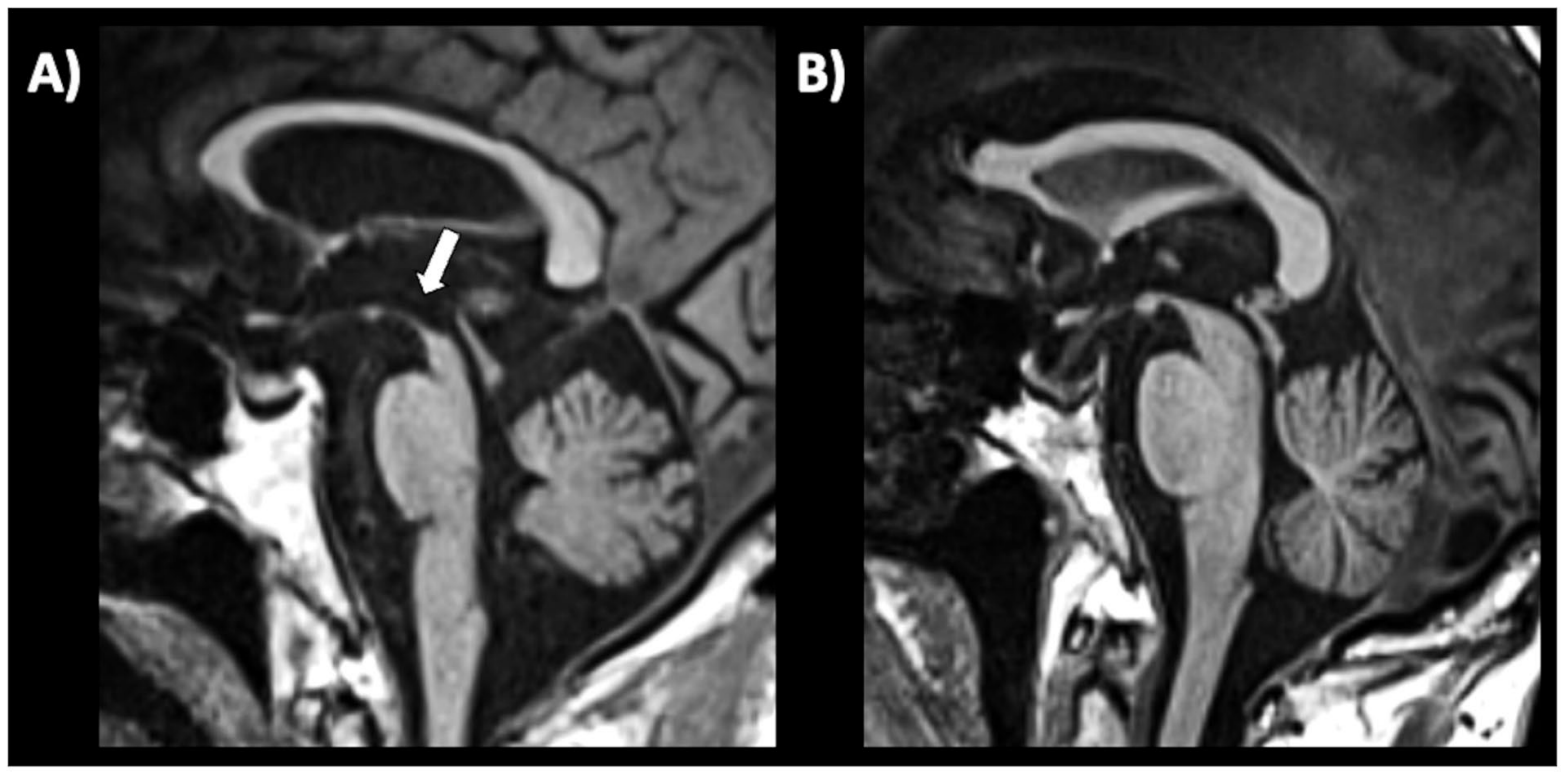
A 3T brain T1-weighted midsagittal MR image showing midbrain atrophy with hummingbird sign in a patient with progressive supranuclear palsy **(A)** and the normal midbrain appearance in a patient with Parkinson’s disease **(B)**. A concave midbrain tegmentum is visible in the image on the left, forming the “beak” of the hummingbird.

Several qualitative radiological signs have been identified for differentiation between PD and atypical parkinsonian disorders. The sensitivity and specificity of these MRI signs, however, are variable and depend on the disease stage, ranging from reasonably good performance in advanced stages to rather poor at the beginning of the disease. Some of the most recognized signs include the “hummingbird” sign (flat or concave midbrain tegmentum [beak] with preserved pontine volume [body] in the sagittal plane forming the silhouette of the head of a hummingbird or king penguin), shown in Figure 5 (184), the “morning glory flower” sign (reduced anteroposterior midbrain diameter with concavity of the lateral margin of the midbrain tegmentum in the axial plane resembling a lateral view of the morning glory flower) (185) and the “Mickey Mouse” sign characterized on axial views by reduction in anteroposterior diameter of the midbrain and thinning of the cerebral peduncles (186, 187) supporting a diagnosis of progressive supranuclear palsy (PSP). In a large cohort of 481 patients with neurodegenerative parkinsonism (85 PSP, 289 PD and 97 MSA) and 79 healthy controls, the hummingbird sign was found in 55.3% of PSP patients and in <1% of PD patients, MSA patients and healthy controls (specificity >99%) (188). The presence of morning glory flower sign yielded a similar high specificity but showed even lower sensitivity (37.7%) for a diagnosis of PSP compared with non-PSP parkinsonism (PD and MSA) (188). In addition, both signs showed sensitivity of 35.3% in early clinically unclassifiable parkinsonism (188). Very recently, some authors compared the accuracy of different qualitative MRI signs (hummingbird, Mickey mouse, and Morning-glory signs) in differentiating PSP-Richardson’s syndrome (PSP-RS) and other PSP variants from healthy controls, showing that all these MRI signs had significantly better performances in identifying PSP-RS cases than PSP variants (189).

##### 4.1.2.2 PD vs secondary parkinsonism

PD and normal pressure hydrocephalus (NPH) may share clinical signs such as parkinsonism, cognitive decline, gait disorders and urinary urgency. Differentiating these two diseases only on clinical basis may be at times challenging and MRI and spinal tap can be necessary for an accurate differential diagnosis. Radiologically, NPH is considered a PSP mimic because of the presence of small midbrain, possibly due to compression by the enlarged third ventricle. On these bases it is possible to hypothesize that qualitative MRI signs such as hummingbird, morning glory, and mickey mouse signs might also help clinicians to differentiate between PD and NPH patients. At present time, however, no study has directly investigated the performances of these qualitative MRI biomarkers in differentiating between PD and NPH. On the contrary, several studies investigated the possible role of hummingbird, mickey mouse, and morning-glory sign in differentiating PSP from NPH. Some authors (51) reported high percentages of patients showing hummingbird sign in both PSP and NPH cohorts confirming that these two diseases share common MRI features. Other authors (50) compared all the three qualitative signs of midbrain involvement in distinguishing between PSP and NPH, demonstrating that Morning glory sign and Mickey mouse sign were more specific for PSP (95%) than hummingbird sign, but lacked sensitivity (14 and 23%, respectively). More recently, Virhammar et al. (190) reported the hummingbird sign in 77% of PSP, in 65% of NPH and in 3% of control subjects, confirming the usefulness of this sign for differentiation of PSP and NPH from controls and the radiological overlap between these two diseases.

#### 4.1.3 Supratentorial (Putaminal rim, hypointensity or atrophy) and infratentorial features (middle cerebellar peduncles atrophy or hyperintensity; pons atrophy, hot-cross bun)

##### 4.1.3.1 MSA-P

###### 4.1.3.1.1 Hyperintense putaminal rim

*Hyperintense putaminal rim* on 1.5T T2-weighted MR images was originally described in MSA patients, and studies reported good performances in distinguishing MSA from PD (191, 192). On 3T MR images, however, it seems to be a nonspecific finding observed not only in patients with MSA, but also in PD and healthy subjects though with milder hyperintensity than in MSA patients; for this reason, it has not been included among imaging feature supportive of MSA in the most recent diagnostic criteria (193).

###### 4.1.3.1.2 Putaminal hypointensity

*Putaminal hypointensity* is a useful sign to differentiate MSA-P from PD patients (193–195). One study comparing several MR qualitative signs, reported high sensitivity (88.9%) with a lower specificity (70%) for putaminal hypointensity in distinguishing MSA-P from PD (192). A recent meta-analysis showed that putaminal hypointensities on T2*-weighted gradient echo (T2* GRE) or susceptibility-weighted imaging (SWI) had pooled sensitivity of 65% (95% CI 51–78%) and specificity of 90% (95 CI 83–95%) in distinguishing MSA-P from PD (196).

###### 4.1.3.1.3 Putaminal atrophy

**Figure 6:**
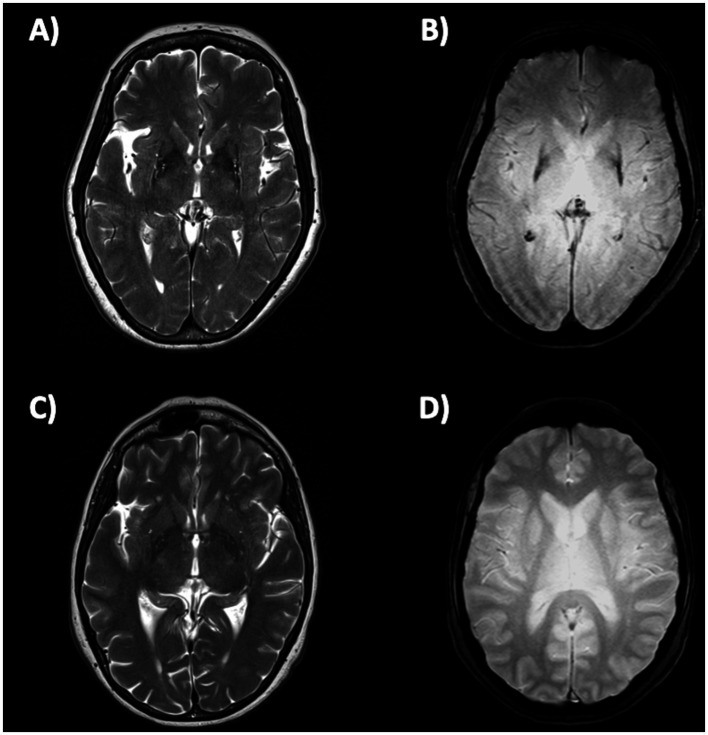
Brain axial 3T-MR images showing atrophy **(A)** and hypointensity **(B)** of postero-lateral putamina in multiple system atrophy—parkinsonian type patient, and the normal putaminal appearance in a patient with Parkinson’s disease **(C,D)**. Figures **(A–C)** are T2-weighted axial MR images; figures **(B–D)** are T2*-weighted gradient echo (T2* GRE) axial MR images.

*Putaminal atrophy* on T1-weighted images showed a sensitivity of 83% and a specificity of 87% in distinguishing MSA-P from PD in one study (192), while other authors reported lower sensitivity, around 50% (197–199). This radiological sign, however, is not easy to evaluate and measure on MR images. Putaminal alterations such as atrophy, hypointensity and putaminal rim on T2-weigthed images May also precede clinical diagnosis in about 30% of patients with MSA-P, potentially contributing to an early diagnosis of the disease (200, 201). Putaminal atrophy and hypointensity MRI signs are shown in Figure 6.

##### 4.1.3.2 MSA-C

**Figure 7:**
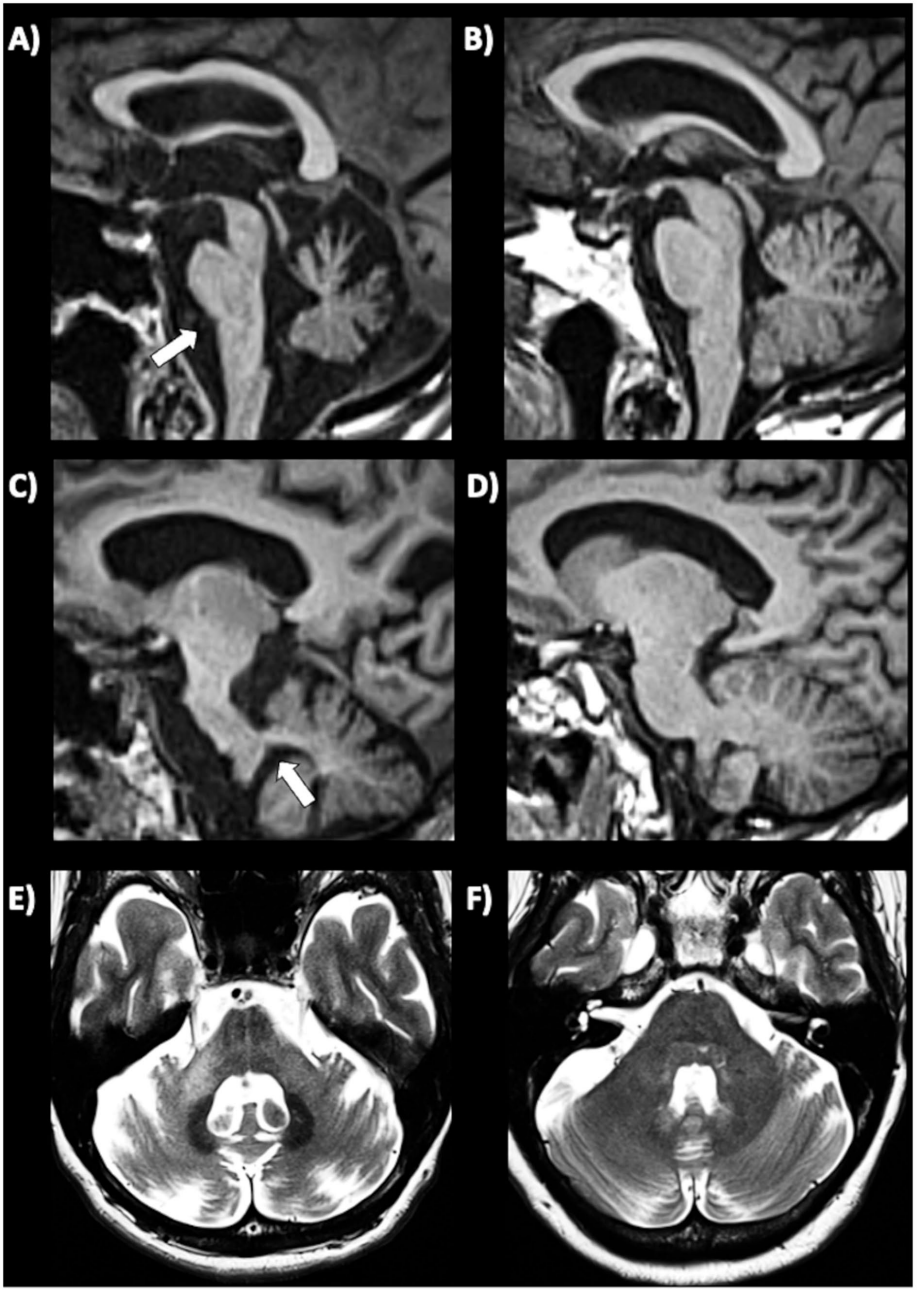
Brain axial 3T-MR images showing infratentorial atrophy in multiple system atrophy. Midsagittal and parasagittal T1-weighted MR images, respectively, show pons atrophy **(A)** and middle cerebellar peduncle atrophy **(C)** (highlighted by the white arrows) in a patient with MSA-C, and the normal pons and middle cerebellar peduncle appearance in a patient with PD **(B–D)**. A T2-weighted axial MR image **(E)** show a cruciform hyperintensity in the pons (“hot cross bun” sign) in an MSA-C patient, and absence of this sign in a PD patient **(F)**. MSA-C, multiple system atrophy—cerebellar type; PD, Parkinson’s disease.

In *MSA-C* patients, several infratentorial features such as *hyperintensity or atrophy of the middle cerebellar peduncle (MCP)* and *atrophy of cerebellum and pons* and *hot-cross bun sign* had strong discriminating power comparing to PD and healthy controls (192, 193, 202–204). In MSA-C, the most sensitive findings were atrophy of MCP and pontine atrophy (100%, both) (Figures 7A–D) while signal increase in the MCP and hot-cross bun showed the highest specificity (100%) (192). The hot cross bun sign (HCBs) is a cruciform configuration of hyperintensity in the pons on T2-weighted MR images attributed to degeneration of transverse pontocerebellar fibers (Figures 7E,F). Despite being a radiological hallmark for MSA (205) with high specificity (96.7%), its sensitivity is only 37–50% (192, 198). Some authors (206) investigated 81 MSA patients (50 MSA-C and 31 MSA-P) with HCBs demonstrating that the severity of this sign showed a positive linear correlation with the scale for assessment and rating of ataxia scores in MSA-C, suggesting that HCBs is a potential imaging marker for the severity of cerebellar ataxia (206). The increase in the HCBs grade was associated with an increased likelihood of disability in MSA-C, but not MSA-P cases, suggesting that it may be a useful imaging indicator for disease progression in patients with MSA-C (206). Although the HCBs is typically considered pathognomonic for MSA in the context of degenerative parkinsonian syndromes, a note of caution is needed in clinic due to the possible radiological overlap between MSA-C and other conditions, such as spinocerebellar ataxia (SCA) type 2 (207, 208). Among the different forms of SCA, HCBs is quite rare in SCA 1, 3, 6, 7, 8, while it has been consistently reported in SCA 2 (209–212), with a prevalence ranging from 10–12% in early-stage patients to around 30% of patients in later disease stages (212).

##### 4.1.3.3 MSA-P vs. MSA-C

MSA-P vs. MSA-C: supratentorial parameters were more frequent in MSA-P but were not valid to differentiate between MSA-P and MSA-C, except for putaminal atrophy. Among the infratentorial features, pons atrophy, signal increase or middle cerebellar peduncle atrophy, hot-cross bun sign were more frequent in MSA-C, and were good for distinguishing MSA-C- from MSA-P (192).

### 4.2 Simple quantitative linear MRI measures

Simple quantitative linear MRI measures (morphometry) which can be manually performed on MR images can provide valuable help in the differential diagnosis of parkinsonism, including Parkinson’s disease and other parkinsonian disorders (151, 155, 213). Quantitative measures offer several advantages in respect to qualitative MRI signs, providing objective numerical data and reducing the reliance on subjective interpretations. They offer measurable parameters that can be compared across individuals or tracked over time, aiding in diagnostic accuracy, and monitoring disease progression. These MRI measures may detect subtle early changes in the brain enabling prompt detection and intervention, supporting clinicians in the early diagnosis of parkinsonian syndromes when symptoms may be mild or ambiguous. Despite their advantages, there are some limitations to consider:

a) Some quantitative MRI techniques require specialized imaging protocols, equipment, or expertise, which may not be widely available in all healthcare settings.

b) Most quantitative linear MRI measures assess width or diameter of small brain structures, such as midbrain, pons or cerebellar peduncles, which typically are in the range of a few millimeters. In this context, differentiating normal variations from subtle pathological changes may be challenging, and there may be inter-observer variability in the measurements and interpretation.

c) Quantitative MRI measures can be more time-consuming compared to conventional qualitative MRI, potentially affecting the feasibility of their routine clinical use.

The most common simple quantitative MRI measurements are linear measures of brainstem structures and third ventricle. These single measurements can also be combined to better differentiate between PD and atypical parkinsonism.

#### 4.2.1 Midbrain diameter and midbrain-to-pons diameter ratio

Several linear brainstem measures have been proposed for differentiating between PD and other neurodegenerative parkinsonism. Among these, the most used ones are the midbrain diameter and the midbrain-to-pons diameter ratio.

**Figure 8:**
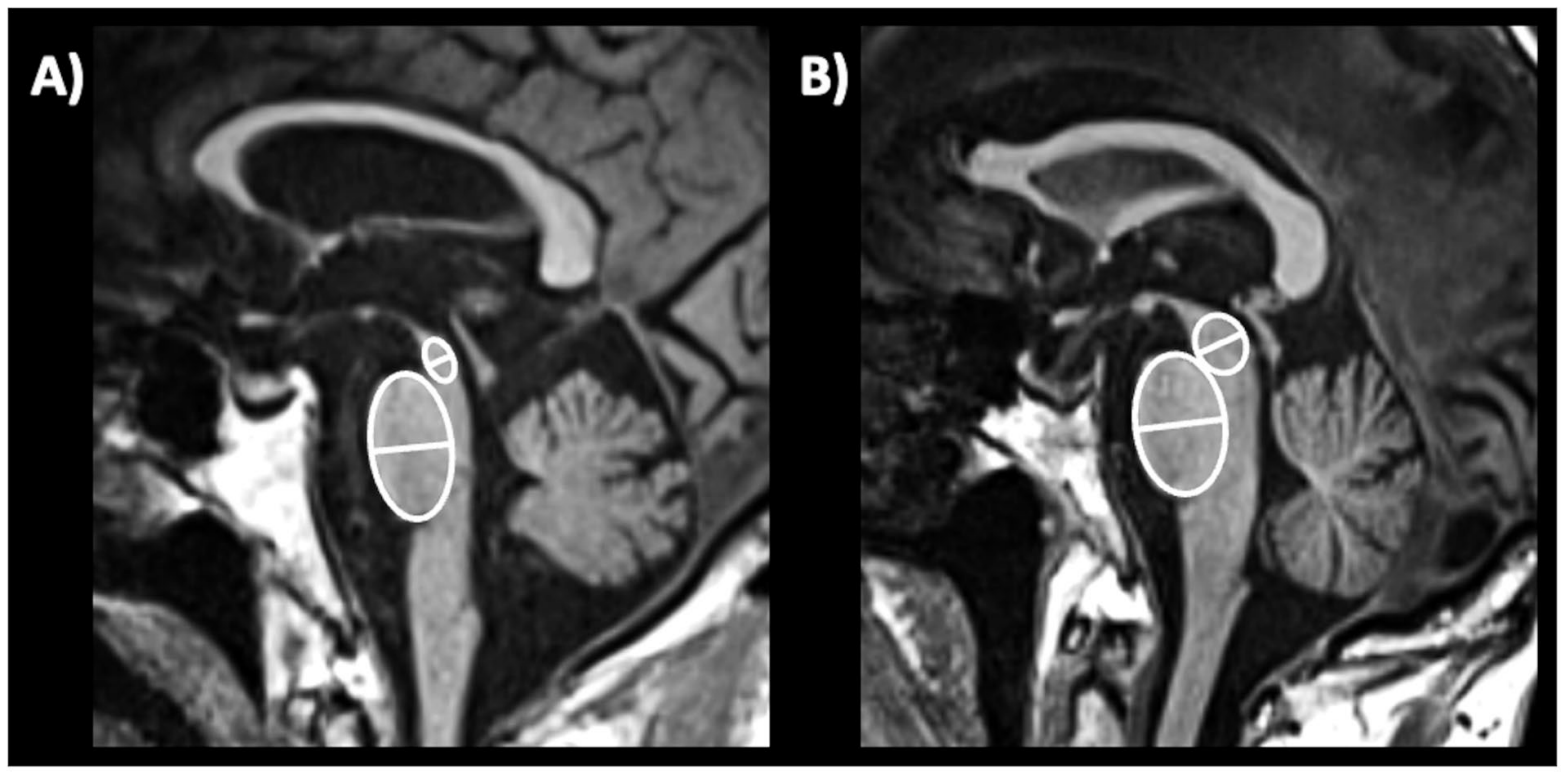
A 3T brain T1-weighted midsagittal MR image showing how to measure the midbrain and pons diameters, as described by Massey et al. (221) in a patient with progressive supranuclear palsy **(A)** and in a patient with Parkinson’s disease **(B)**.

a) *Midbrain diameter*. In 2001, Warmuth-Metz and colleagues described the measurement of midsagittal anteroposterior diameter (AP) of some brainstem structures including midbrain, pons and collicular plate in 50 patients with various parkinsonian syndromes (20 PD, PSP, 14 MSA,16 PSP) and 12 control subjects (214). The authors found that midbrain diameters of PSP patients were lower than those of PD and control subjects concluding that anteroposterior diameter of the midbrain on T2-weighted magnetic resonance may be a reliable measure to differentiate PSP patients from patients with PD *in vivo*. On the contrary, the midbrain diameter values had large overlap between MSA and PD patients, resulting not helpful to support the differential diagnosis between these two diseases (214). Several other studies have investigated the accuracy of midbrain diameter measured on axial or sagittal MRI planes (215–219). Among the largest studies, some authors (219) showed that a midsagittal midbrain diameter <8.9 mm optimally separated PSP (AUC 0.90) from non-PSP patients while it was less accurate in differentiating MSA from non-MSA (AUC 0.50) and PD from non-PD (AUC 0.78). A more recent study comparing different PSP subtypes (220) showed that the sagittal midbrain diameter was lower in PSP-RS (7.60 ± 1.08 mm) than in PSP-P (8.43 ± 1.30 mm), and that this measure differentiated PSP-P from PD patients (10.21 ± 0.89) with AUC 0.87 (220). Conversely, the midbrain diameter failed in distinguishing PSP variants (other than -RS) from controls, due to large overlap in another study, leading to mixed results in PSP variants due to the commonly observed lower degree of midbrain atrophy (189). A study carried out in a small cohort of patients with pathologically confirmed PSP, PD and MSA patients confirmed that midsagittal midbrain diameter was reduced in PSP patients (8.1 ± 1.2) in comparison with PD, MSA and controls (221). In pathologically confirmed cases a midbrain diameter of <9.35 had 83% sensitivity, and 100% specificity in differentiating PSP from non-PSP patients (221). The strength of this study was the inclusion of patients with pathologically confirmed diagnosis, but the limitation was the small sample size, thus requiring further studies to confirm these findings. Overall, midbrain diameter seems a useful measure for differentiating PSP-RS from other neurodegenerative parkinsonism. However, most of these studies were performed in small samples of patients with different neurodegenerative disorders and further validation measurements in larger cohorts of patients are needed to confirm these findings.

b) The “*One line*” method is a recent linear measure of brainstem size calculated as the length of the longitudinal axis of midbrain and pons on the mid-sagittal view (222). This method was tested in 101 subjects who underwent 3.0T MRI (20 controls, 44 PD, 20 MSA, 12 PSP and 5 corticobasal syndrome). The authors found that their “one line” method was comparable with other areas measures or combined indexes such as the MR Parkinsonism index (223, 224) in terms of accuracy of diagnosis but was preferable due to a faster processing speed. The limitations of this method were the small sample size of each patient group and the lack of a validation cohort, requiring further studies before using this measure in clinical practice.

c) *Sagittal midbrain-to-pons diameter ratio (Md/Pd)*: in 2013, Massey et al. proposed the ratio between midbrain diameter and pons diameter (Figure 8) as a simple biomarker to distinguish among parkinsonian syndromes (221). In a couple of small studies on pathologically confirmed cases, the Md/Pd showed was lower in PSP (0.47 ± 0.08) than in PD (0.57 ± 0.05), MSA (0.70 ± 0.11) and controls (0.63 ± 0.03), and a threshold of 0.52 for the midbrain-to-pons ratio had a specificity of 100% but a sensitivity of 67% for differentiating PSP from non-PSP patients (221, 225). Other authors (219) demonstrated that a similar value of midbrain to pons ratio (<0.54) was accurate (AUC 0.93) in differentiating PSP from non-PSP while was less accurate (AUC 0.71) in distinguishing MSA from non-MSA (0.71) and PD from non-PD (AUC 0.63). One recent large study also showed that a ratio ≤0.56 had potential for distinguishing PSP-P from PD (sensitivity: 86.0%, specificity: 90.7%) (220). A recent meta-analysis on conventional magnetic resonance imaging in diagnosis of parkinsonian disorders, however, pointed at the midbrain diameter as the most powerful simple linear measure to distinguish between PSP and PD more than Md/Pd (204); thus, the superiority of Md/Pd over the midbrain diameter alone needs to be further investigated.

d) Decisional tree algorithm is a model to improve the diagnostic accuracy of specific linear measures which have suboptimal performances when used alone. Some authors (219) investigated decisional tree algorithm using linear measures for differentiating among PD and atypical parkinsonism (PSP and MSA) and reported good classification performances also in patients with unclassifiable parkinsonism (with available clinical follow-up) (219). When decision tree algorithm was applied for the differential diagnosis between PSP and non-PSP, Md/Pd ratio combined with midbrain diameter values yielded high sensitivity (AUC 0.90), while the accuracy of this model was suboptimal in discriminating between PD and MSA (219).

#### 4.2.2 Pons diameter

A few studies in small cohorts of PD and MSA patients found that pons atrophy was more frequent in MSA than in PD patients, with smaller pons diameter (191, 204, 219, 226) and area (223, 227). A very recent study (228) in a large cohort of 137 MSA-C patients confirmed that the antero-posterior pons diameter was reduced in MSA-C than in PD, MSA-P and other atypical parkinsonism, also showing greater reduction over time. However, the real accuracy of this simple measure to distinguish MSA-C and especially MSA-P from PD remains largely unexplored.

#### 4.2.3 Middle cerebellar peduncle diameter

**Figure 9:**
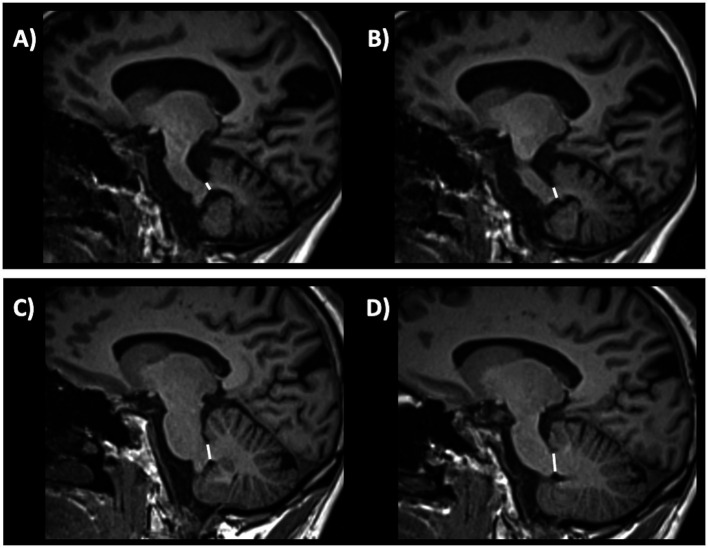
Three-tesla brain T1-weighted parasagittal MR images showing how to measure the middle cerebellar peduncle width measurements, in a patient with multiple system atrophy **(A,B)** and in a patient with Parkinson’s disease **(C,D)**. This structure is more atrophic in multiple system atrophy than in Parkinson’s disease.

The middle cerebellar peduncle (MCP) is a fiber pathway involved in motor coordination connecting the cerebellum to the pons. In 2006, a study (229) first reported in a small cohort that middle cerebellar peduncle (MCP) width measured on parasagittal MR images at cut off ≤8.0 mm accurately separated (sensitivity and specificity 100%) MSA from PD (Figure 9). These findings were subsequently confirmed by other studies showing that MCP width was significantly smaller in MSA relative to PD (228, 230–232), and others found that MCP diameter accurately differentiated (AUC 0.84) MSA from non-MSA patients with a cut-off <8 mm (219).

#### 4.2.4 Superior cerebellar peduncle diameter

**Figure 10:**
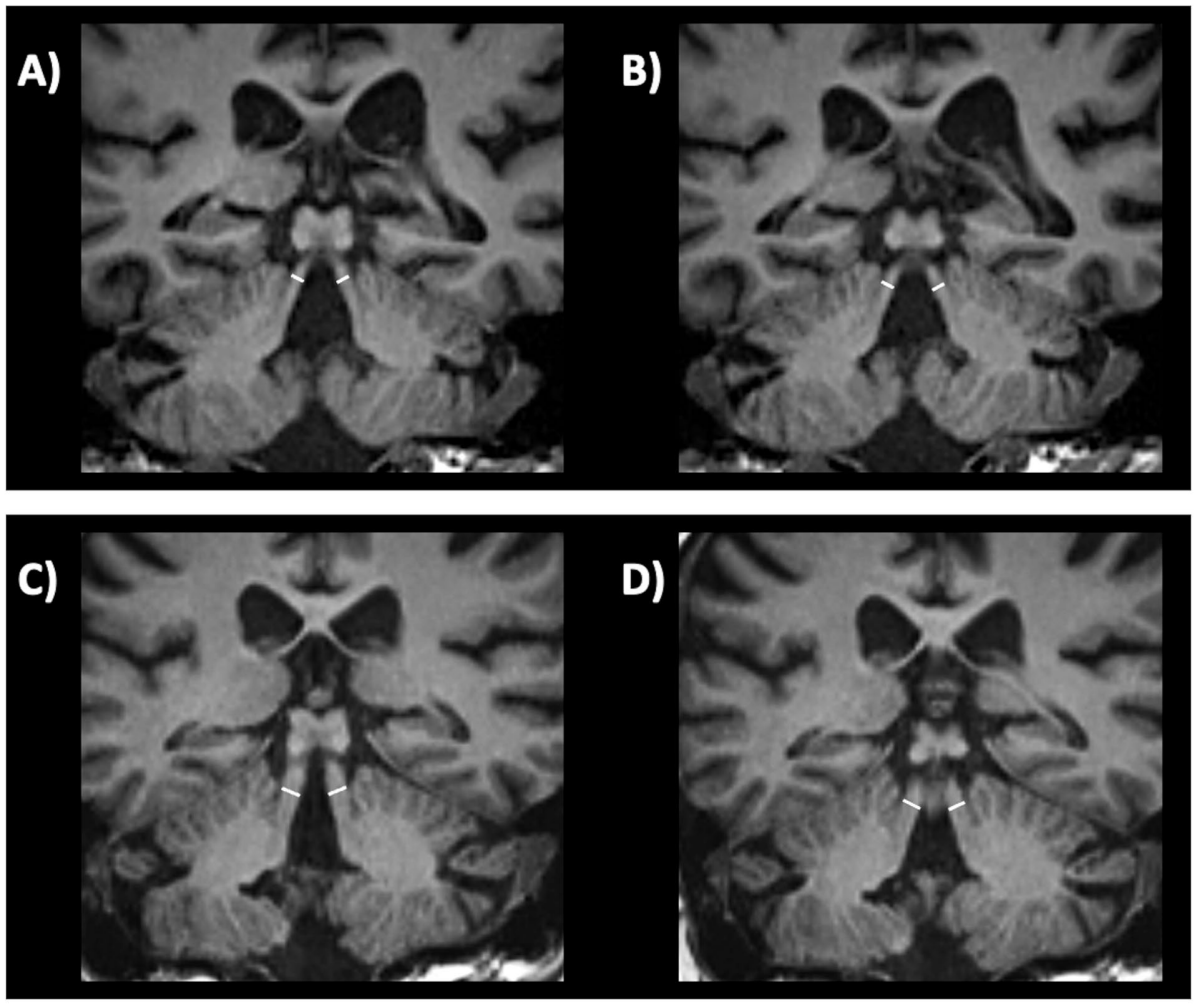
Three-tesla brain T1-weighted oblique coronal MR images parallel to the floor of the fourth ventricle, showing how to measure the superior cerebellar peduncle width, in a patient with progressive supranuclear palsy **(A,B)** and in a patient with Parkinson’s disease **(C,D)**. This structure is more atrophic in progressive supranuclear palsy than in Parkinson’s disease.

MRI measurements can be used to evaluate the integrity of the superior cerebellar peduncle (SCP). The SCP width (diameter) is typically measured on oblique coronal T1-weighted MR images (parallel to the floor of the fourth ventricle), as shown in Figure 10, and may provide useful information for evaluating this small brain structure which is specifically atrophied in patients with PSP (233, 234). In 2008, a study (223) first demonstrated that the SCP width was significantly reduced in patients with PSP compared to patients with MSA and PD. Another study (218) carried out in a small sample of patients with PSP (*n* = 24), MSA-P (*n* = 9), PD (*n* = 18) and controls (15) showed that the SCP by midbrain product provided an excellent combination of sensitivity (100%) and specificity (98%) for diagnosis of PSP. A meta-analysis overall confirmed the usefulness of SCP measurement but highlighted that there was some heterogeneity in terms of SCP size reduction in patients with PSP compared to those with PD; on the other hand, there was homogeneous agreement that the SCP was smaller in patients with PSP compared to those with MSA (235). The SCP width is usually smaller in PSP-RS than in PSP-P patients (224, 236). The manual measurement of SCP diameter (or width) is not easy and should be especially employed in research centers due to a large variability of the manual measures which can be observed in the same patients with low inter-rater agreement (237). Automated measurements of the SCP width (224, 238) have strongly improved the feasibility and reproducibility but this technique is not widely available yet for clinical practice.

#### 4.2.5 Length of tectal plate

**Figure 11:**
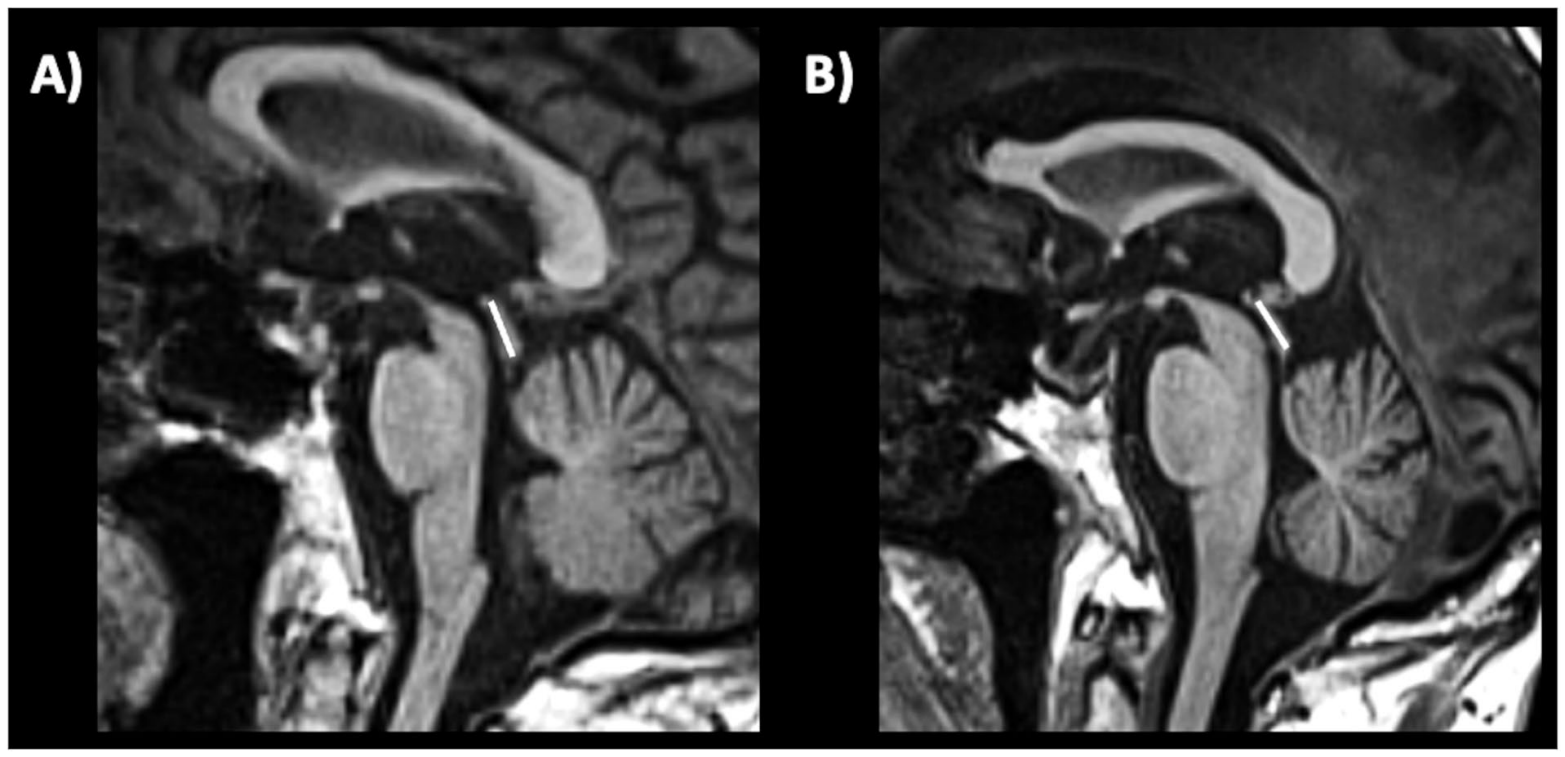
A 3T brain T1-weighted midsagittal MR image showing how to measure the length of the tectal plate, in a patient with progressive supranuclear palsy **(A)** and in a patient with Parkinson’s disease **(B)**.

Very recently, a preliminary study (239) found that the length of the tectal plate, measured on midagittal MR images (Figure 11), was reduced in both PSP-RS (*n* = 20) and PSP-P (*n* = 20) compared with PD and control subjects. The tectal plate length showed good potential in differentiation of PSP-RS from controls (AUC 0.87) and PD (AUC 0.90) while a moderate differentiation of PSP-P from controls (AUC 0.79) and PD (AUC 0.78) was obtained with limited specificity (65%). On the contrary, the tegmental plate width was not useful for classification purposes (239). Future studies are needed to compare this new potential biomarker with other linear measures of brainstem regions.

#### 4.2.6 Third ventricle diameter

**Figure 12:**
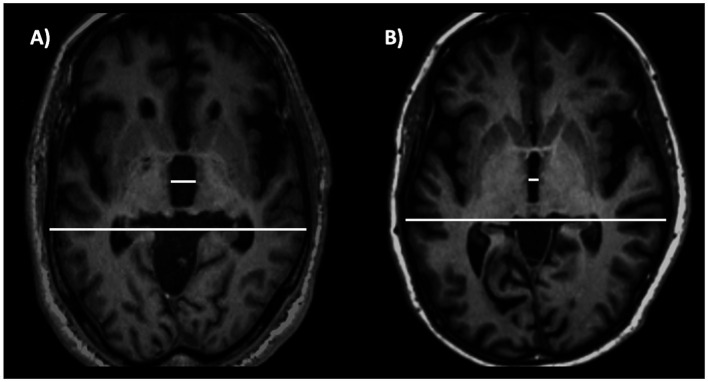
A 3T brain T1-weighted axial MR image at the level of the third ventricle’s maximum dilation, showing how to measure the third ventricle width. This measure is normalized by the internal skull diameter on the same axial slice. The figure shows this measure in a patient with progressive supranuclear palsy **(A)** and in a patient with Parkinson’s disease **(B)**. This structure is enlarged in progressive supranuclear palsy compared to Parkinson’s disease.

The third ventricle has been consistently reported to be enlarged in PSP, by sonographic and MRI studies (131, 240–242). A recent large study (243) involving two independent cohorts evaluated the usefulness of measuring the third ventricle width on axial MR images in PD and PSP patients, demonstrating that the third ventricle width (normalized by the internal skull diameter on the same axial slice) accurately differentiated between PD and PSP (Figure 12), also in the early stages of the diseases. In addition, some authors (244) demonstrated that the third ventricle width was significantly associated with higher risk of dependency on wheelchair in PSP-RS patients, suggesting a prognostic value of this simple MR measure. Differently from most of the brainstem measures described above, which are smaller (reflecting more severe atrophy) in PSP-RS than PSP-P, the third ventricle may be similarly enlarged in these two PSP subtypes (220, 245), making this sign potentially useful for differentiation between PSP-P and PD (220). No data are currently available in MSA patients. The great advantage of using this MR biomarker is its simplicity and generalizability that make this measure ideal for diagnostic purposes in clinical setting.

### 4.3 Limitations of the available evidence

Among the plethora of biomarkers proposed over the last couple of decades, a few diagnostic tests showed the prerequisites to be used for supporting neurologists in the differential diagnosis of PD in routine clinical practice, mainly limited cost and expertise required, availability, and classification accuracy. None of the biomarkers presented in this comprehensive review, however, is currently included in PD criteria (2) though some time has passed since when these biomarkers were first proposed, highlighting a gap between research discoveries and integration of this knowledge into clinical practice. One of the main reasons is the lack of enough data to clearly establish the role of these biomarkers in PD diagnosis, due to the limitations of the available evidence. For some of these biomarkers, such as the BRrc, the rest tremor pattern and several MR measurements, most evidence came from small studies from different centres with overall promising findings but large heterogeneity in the methods and results, or from large single-centre studies lacking validation. For nigrosome-1 sign and transcranial sonography, evidence converge on the differential diagnosis between PD and healthy subjects, which is generally not a very difficult challenge (apart for PD patients in a very early stage showing subtle or ambiguous clinical signs), but studies investigating the usefulness of these signs in distinguishing PD from other diseases are limited and often showed suboptimal accuracy, meaning that these signs can be observed also in other parkinsonian syndromes, requiring a note of caution in the interpretation and a reflection on the best scenario for their appropriate clinical application. On the other hand, MRI biomarkers aiming to distinguish between PD and other parkinsonism are often based on the presence of atrophy of specific regions in other diseases but not in PD, thus not representing imaging biomarkers “specific of PD” and not allowing to distinguish PD from healthy controls. For all considered biomarkers, most evidence came from studies carried out in Europe or North America, and data in under-represented population from low-income countries are currently lacking. Finally, many of the discussed biomarkers have been investigated in terms of a binary response (normal or abnormal), which may limit their ability to reflect how advanced the disease is and track disease progression.

### 4.4 Future directions

Based on the gaps and limitations discussed above, the first future challenge would be to provide robust evidence on the diagnostic potential of these measures in larger international cohorts of PD patients and atypical parkinsonian or tremor syndromes to improve the generalizability of the results. The recent huge efforts of the community to promote international initiatives for collecting multimodal data of patients with PD (i.e., the Parkinson’s Disease Progression Marker Initiative, PPMI) (246) and other diseases are well aligned with these needs and may significantly impact the research in this field, providing invaluable resources to investigate biomarker usefulness in large multicentre cohort. This can be especially true for MRI-based biomarkers in the near future, because of well-established imaging protocols and harmonization procedures currently available aiming to obtain comparable data across centres; on the other hand, future efforts may be directed to the development of standardized protocols for electrophysiological data collection (i.e., tremor analysis) to build new data resources for the development or validation of promising biomarkers.

A second point definitely needing future research is the development of simple biomarkers aiming not only to support PD clinical diagnosis, but also to identify patients at risk of developing PD, to stratify PD patients into subtypes with similar prognosis, and to track or predict disease progression, which are all tasks of pivotal importance for both clinical trials and patient care (24). In addition, PD is moving from a clinical to a biological entity (247, 248), requiring the use of highly complex technologies to provide molecular evidence of synucleinopathy, neurodegeneration or genetic mutations. The development of accurate and validated simple biomarkers to predict either one or more of these PD features supporting the clinical diagnosis would be of extreme relevance. It remains to be clarified if and to what extent any of the simple biomarkers proposed in this review may serve to these aims, and future studies are needed to clarify these points.

## 5 Conclusion

In clinical practice, the diagnosis of parkinsonism, including Parkinson’s disease and other Parkinsonian syndromes, is primarily based on clinical evaluation and medical history. However, electrophysiological parameters and conventional MRI (qualitative assessment and simple linear measurements) can be helpful tools to support the clinical diagnosis by ruling out other diseases potentially mimicking PD (Table 1). Biomarkers for routine clinical practice need to be simple, accurate and not too expensive, usually being electrophysiology or MRI based. The most important electrophysiological measures include R2-BRrc and tremor pattern whereas T1-MRI based measures include qualitative (Hummingbird, morning glory and Mikey mouse signs) and quantitative measures such as midbrain diameter, midbrain-to-pons diameter ratio, middle and superior cerebellar peduncles diameter and third ventricle width, that can be used alone or included in decisional tree algorithms to support PD differential diagnosis. Advanced imaging techniques and nuclear medicine technology including PET and SPECT are useful to support the differential diagnosis between PD and other diseases. The SPECT with 123I-ioflupane (DaTscan) has been approved from the Food and Drug Administration more than twenty years ago for differentiating PD from ET, and has a crucial role in differentiating TD-PD from other tremulous diseases often leading to changes in the clinical diagnosis and therapeutic approach (17, 71, 249). On the other hand, PET imaging with 18F-fluorodeoxyglucose (FDG-PET) is a valid tool to help clinicians in the differential diagnosis between akinetic-rigid PD and atypical parkinsonism such as PSP, MSA or CBD, due to the presence of different disease-specific metabolism pattern (i.e., striatal hypermetabolism and/or parietal hypometabolism in PD, frontal hypometabolism in PSP, putaminal hypometabolism in MSA) (250–253). These techniques, however, rely on a nuclear medicine unit and thus May suffer from limited availability worldwide; in addition, FDG-PET imaging achieves better classification performance when combined with modern deep learning or covariance pattern analyses rather than visual interpretation (254, 255), making these powerful but complex diagnostic procedures out of the scope of the current review. Finally, blood-based biomarkers may also be suitable for supporting PD diagnosis in the future; at present time, however, the most widely investigated or promising biomarkers so far require technologies such as single molecule array (SIMOA), seed amplification assay (SAA), proximity extension assay (Olink) or the identification of neuronally-derived exosomes (256–260), which are expensive and not widely available in routine clinical practice yet. It is possible to hypothesize that future technical standardization and advancement may increase the availability of some of these techniques also in clinical settings, further improving biomarker-assisted PD diagnosis worldwide. In addition, it is possible to speculate on diagnostic strategies for PD combining simple and complex biomarkers, aiming to achieve a reliable PD diagnosis in all setting though simple and available biomarkers supporting the clinical differential diagnosis, and adding information from molecular or genetic markers for a better patient stratification, reducing PD heterogeneity.

**Figure 13:**
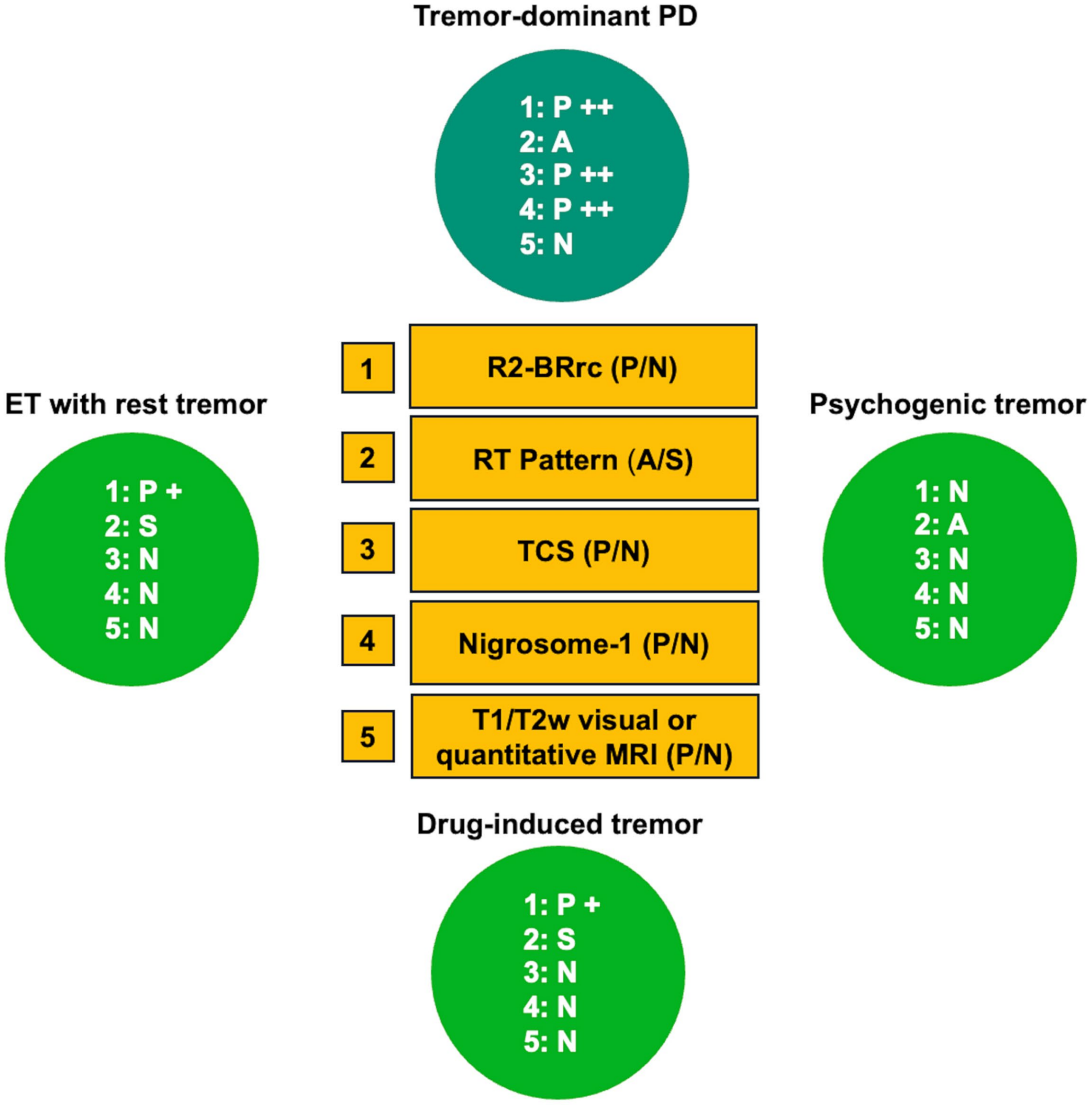
Role of simple biomarkers to distinguishing tremor-dominant PD from other rest tremor syndromes. A grading system (+ or ++) was employed to reflect the degree and frequency of test abnormality. Please note that, despite it is reasonable to hypothesize that ET patients with rest tremor and psychogenic tremor patients have normal substantia nigra echogenicity and normal nigrosome-1 appearance, no data exist on this point. PD, Parkinson’s disease; ET, essential tremor; A, alternating rest tremor pattern; S, synchronous rest tremor pattern; P, pathologic (abnormal); N, normal; TCS, transcranial sonography assessment of substantia nigra echogenicity.

**Figure 14:**
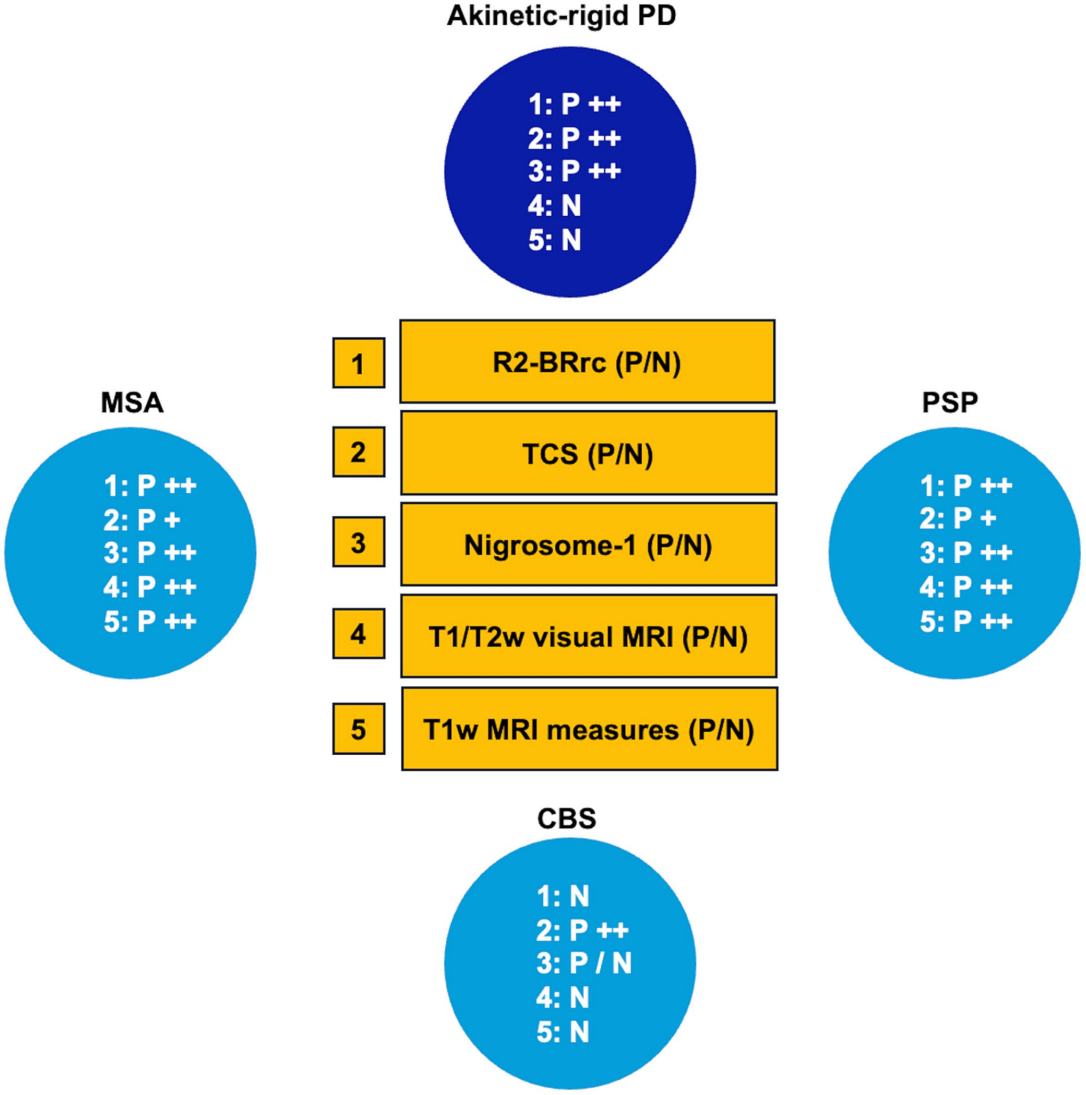
Role of simple biomarkers in distinguishing between rigid-akinetic PD and atypical parkinsonisms. A grading system (+ or ++) was employed to reflect the degree and frequency of test abnormality. P/N label has been assigned to nigrosome-1 evaluation in CBS, since no data exist on this topic. PD, Parkinson’s disease; PSP, progressive supranuclear palsy; MSA, multiple system atrophy; TCS, transcranial sonography assessment of substantia nigra echogenicity; R2-BRrc, R2 component of the blink reflex recovery cycle; P, pathologic (abnormal); N, normal.

Overall, in this review, we included an up-to-date summary on the role of simple biomarkers in distinguishing between tremor-dominant PD and other rest tremor syndromes, and between rigid-akinetic PD and atypical parkinsonism (Figures 13, 14, respectively), and provided evidence-based guidelines for using currently available simple biomarkers in clinical practice for differentiating Parkinson’s disease from parkinsonism, essential tremor, or healthy subjects, as following:

- R2-BRrc can be useful for distinguishing patients with *de novo* Parkinson’s disease from patients with ET, ET plus and healthy subjects.
- Rest tremor pattern can be useful for distinguishing tremulous disorders with striatal dopaminergic damage (typically PD) from tremulous disorders with integrity of dopaminergic system (typically ET plus).
- Transcranial sonography (TCS) may accurately differentiate PD from CBS and control subjects, while it is less accurate in distinguishing PD from neurodegenerative parkinsonism such as PSP and MSA.
- MRI qualitative features:
  -Hummingbird, morning glory and mickey mouse signs have high specificity but low sensitivity for distinguishing PSP from PD.
  -The nigrosome-1 sign: unilateral or bilateral absence of this MRI feature accurately differentiates PD from healthy subjects, also at the early-stage of the disease.
  -Supratentorial features (putaminal atrophy and/or putamen hypointensity), are useful for differentiation of MSA-P from PD.
  -Infratentorial atrophy signs (hot cross-bun, pons and middle cerebellar peduncles atrophy) may be useful for differentiating MSA-C from other atypical parkinsonian syndromes, PD and normal aging controls with high specificity, but low sensitivity.
- MRI quantitative measurements:
  -Midbrain diameter, midbrain to pons diameter ratio, and tectal plate length are simple linear measures and can be useful especially for distinguishing PSP from non-PSP patients but are less accurate for differentiating MSA from non-MSA patients or PD from non-PD patients.
  -Middle cerebellar peduncle (MCP) width may be useful for distinguishing MSA from PD.
  -The third ventricle width can be useful for the early differentiation between PSP and PD.
